# Proteomic profiles before and during weight loss: Results from randomized trial of dietary intervention

**DOI:** 10.1038/s41598-020-64636-7

**Published:** 2020-05-13

**Authors:** Sylwia M. Figarska, Joseph Rigdon, Andrea Ganna, Sölve Elmståhl, Lars Lind, Christopher D. Gardner, Erik Ingelsson

**Affiliations:** 1 Department of Medicine, Division of Cardiovascular Medicine, Stanford University School of Medicine, Stanford, CA 94305 USA; 2 Stanford Cardiovascular Institute, Stanford, CA 94305 USA; 3 Stanford Diabetes Research Center, Stanford, CA 94305 USA; 4 Quantitative Sciences Unit, Stanford University School of Medicine, Palo Alto, CA 94304 USA; 5 Program in Medical and Population Genetics, Broad Institute of MIT and Harvard, Cambridge, MA USA; 6 Stanley Center for Psychiatric Research, Broad Institute of MIT and Harvard, Cambridge, MA USA; 7 Analytic and Translational Genetics Unit, Department of Medicine, Massachusetts General Hospital, Boston, MA USA; 8 Department of Clinical Sciences, Division of Geriatric Medicine, Lund University, Malmö University Hospital, Malmö, Sweden; 9 Department of Medical Sciences, Cardiovascular Epidemiology, Uppsala University, Uppsala, Sweden; 10 Stanford Prevention Research Center, Department of Medicine, Stanford University Medical School, Stanford, CA USA; 11 Department of Medical Sciences, Molecular Epidemiology and Science for Life Laboratory, Uppsala University, Uppsala, Sweden

**Keywords:** Biomarkers, Obesity

## Abstract

Inflammatory and cardiovascular biomarkers have been associated with obesity, but little is known about how they change upon dietary intervention and concomitant weight loss. Further, protein biomarkers might be useful for predicting weight loss in overweight and obese individuals. We performed secondary analyses in the Diet Intervention Examining The Factors Interacting with Treatment Success (DIETFITS) randomized intervention trial that included healthy 609 adults (18–50 years old) with BMI 28–40 kg/m^2^, to evaluate associations between circulating protein biomarkers and BMI at baseline, during a weight loss diet intervention, and to assess predictive potential of baseline blood proteins on weight loss. We analyzed 263 plasma proteins at baseline and 6 months into the intervention using the Olink Proteomics CVD II, CVD III and Inflammation arrays. BMI was assessed at baseline, after 3 and 6 months of dietary intervention. At baseline, 102 of the examined inflammatory and cardiovascular biomarkers were associated with BMI (>90% with successful replication in 1,584 overweight/obese individuals from a community-based cohort study) and 130 tracked with weight loss shedding light into the pathophysiology of obesity. However, out of 263 proteins analyzed at baseline, only fibroblast growth factor 21 (FGF-21) predicted weight loss, and none helped individualize dietary assignment.

## Introduction

Obesity, usually defined by body mass index (BMI), is a causal risk factor for cardiovascular and metabolic diseases, such as coronary heart disease, heart failure, ischaemic stroke and type 2 diabetes^1–3^. There is a growing scientific interest in discovery and measurement of circulating biomarkers linked to obesity and cardiometabolic risk since they may influence health outcomes in several ways. For example, such biomarkers can shed a new light on pathophysiologic pathways, possibly improve identification of subjects at disease risk, and help monitor disease progression and prognosis^4^. Moreover, circulating biomarkers are potential targets for interventions such as diet, lifestyle, or drug treatment; and may allow to advance personalized treatment for individual patients^4^. Weight gain in overweight individuals triggers inflammatory responses and activates pathways involved in cardiovascular disease^5^. Dietary interventions aim to reduce fat mass, restore biological function of adipose tissue, and improve metabolic outcomes^6–8^. Yet, the utility of proteomic profiling in relation to weight loss interventions is scarcely studied, and previous studies have been performed in small study samples including between ~20 and 200 subjects^5,8–11^ or did not attempt to utilize proteins for prediction of weight loss and personalized interventions^12–15^. In contrast, the present study is based on a secondary analysis of what was primarily a weight loss diet study involving 609 generally healthy overweight or obese adults without diabetes, randomly assigned to healthy low-fat (HLF) or healthy low-carbohydrate (HLC) diet, in which we analyzed 263 plasma proteins using the Olink Proteomics arrays. Weight loss response in clinical obesity treatment programs is highly variable and we hypothesized that this heterogeneity can be characterized by a distinct plasma proteomic signature^16^. This may open up avenues for incorporation of protein biomarker measurements into weight loss prediction models to help individuals choose a diet for optimal weight loss. Such a precision medicine approach to weight loss has the potential to reduce the burden of illness and disability due to obesity and its related disorders. In the current secondary analysis of a randomized weight loss dietary intervention study, we used three- and six-months post-randomization data from a 12-month dietary intervention. The aims of this study were to: (1) evaluate associations between circulating protein biomarkers and BMI at baseline; (2) study correlations of protein and BMI changes during a weight loss intervention; and (3) to investigate prediction of weight loss using protein biomarkers during the first three months of intervention (Supplementary Fig. 1).

## Methods

### Study populations

#### The diet intervention examining the factors interacting with treatment success (DIETFITS) study

The DIETFITS is a single-site, randomized weight loss trial comparing two diets as described previously^17^. In short, the DIETFITS study included 609 adults recruited from the San Francisco Bay area in 2013–2015, aged 18 to 50 years without diabetes with a body mass index (BMI) between 28 and 40. Participants were randomized to a HLF diet or a HLC diet for 12 months. Health educators delivered a behavior modification intervention to HLF (n = 305) and HLC (n = 304) participants via 22 diet-specific group sessions over 12 months. The sessions focused on approaches to achieve the lowest fat or carbohydrate intake that could be maintained long-term and emphasized diet quality. Thus, participants reduced total fat or digestible carbohydrates intake to 20 g/d during the first 8 weeks. They then slowly added fats or carbohydrates back to their diets in increments of 5 to 15 g/d per week until they reached the lowest level of intake they believed could be maintained indefinitely. There were no explicit instructions for energy (kilocalories) restriction^17^. In the current study, we used data from the baseline, 3- and 6-month time points. For all participants subsequent blood samples were taken at baseline and 6 months via venipuncture at the Stanford’s Clinical and Translational Research Services (CTRU) by trained nurses or phlebotomists. Aliquots of plasma and serum were obtained at both time points and immediately stored in a − 80° freezer until the time of analysis as have been described previously^18^.

#### EpiHealth

The EpiHealth study used for replication for our first aim (cross-sectional associations) was initiated in 2011 recruiting men and women aged 45–75 years in the Swedish towns of Uppsala and Malmö^19^. The present study including 1,584 overweight/obese individuals (BMI ≥ 25 kg/m^2^) with measured protein profiles served as a replication cohort for baseline associations in DIETFITS.

The DIETFITS was approved by the Stanford University Human Subjects Committee and registered at ClinicalTrials.gov (NCT01826591). All study procedures were in accordance with the Helsinki Declaration of 1975 as revised in 1983, and all study participants provided written informed consent. The EpiHealth study was approved by the Ethics Committee of Uppsala University, conducted in compliance with the Declaration of Helsinki, and all subjects gave their informed written consent.

### Proteomic data

Proteomic profiling was performed in 1 µL of plasma samples using the Olink Proteomics CVD II, CVD III and Inflammation assays (http://www.olink.com) simultaneously measuring proteins associated with cardiovascular disease and inflammation. The Inflammation assay was not measured in EpiHealth, thus inflammation biomarkers were not available for replication. The kits are based on the proximity extension assay (PEA) technology, where 92 oligonucleotide-labelled antibody probe pairs are allowed to bind to their respective target present in the sample. The PEA technique has a high specificity and sensitivity^20,21^. The platform provides normalized protein expression (NPX) data where a high protein value corresponds to a high protein concentration, but not an absolute quantification. We included all samples that had valid measurements and replaced values below level of detection (LOD) with values of LOD/2 (as performed in^22^). If more than five proteins failed on a specific panel, that sample was excluded for that panel for further analyses. Moreover, we excluded 3 proteins because all subjects had values below LOD (NT-proBNP, IL-2 and IL-22RA1). Proteins with ≥10% missingness (BNP, CA5A, SLAMF7, IgG Fc receptor II-b) were excluded in EpiHealth.

### Statistical analysis

#### Aim 1: Cross-sectional analyses of protein levels with BMI at baseline

To allow comparison of effect sizes between different proteins, we converted log_2_-transformed protein levels to the SD-scale by rank transformation. Associations between protein levels and BMI at baseline were analyzed using linear regressions adjusted for age, sex and race. Associations with a false discovery rate (FDR) < 5% were considered significant, and *P* < 0.05 with the same direction of association was considered a successful replication in EpiHealth. We have previously shown that this strategy results in a validation FDR < 1%^23,24^.

#### Aim 2: Longitudinal changes in protein levels and BMI during 6 months

To study associations of protein changes over time with BMI changes over time, we used linear mixed effect models including data from baseline and 6 months. Statistical models included the outcome BMI; fixed effects for time, age, sex, race, protein levels; and a random effect for individual. Proteins were analyzed in separate models, and results with FDR < 5% were considered significant.

#### Aim 3: Prediction of weight loss success using circulating proteins at baseline

The goal of these analyses was to assess the predictive value of baseline protein levels for weight loss success beyond simple demographic factors. We focused on the first 3 months as we were interested in the early effects of the dietary intervention – the greatest weight loss occurs in the initial period of a diet – and to maximize statistical power (fewer missing weight measures at 3 months [n = 77] compared to 6 months [n = 138]). We calculated weight loss for each individual as a change in BMI by subtracting values of baseline BMI from BMI at 3 months (ΔBMI = BMI_3months_ − BMI_baseline_). Hence, a negative value in this variable means successful loss of weight. This weight loss variable was used as the dependent variable with each protein as the independent variable in separate linear regression models. Proteins with FDR < 5% were considered significant. Moreover, we tested interaction effects between each protein at baseline and diet assignment on weight loss to explore whether protein biomarkers help individualize dietary assignment in a weight loss program. Proteins were analyzed in separate linear regression models adjusted for diet assignment age, sex race, and the interaction term between diet assignment and protein. Diet by protein interaction terms with FDR < 5% were considered significant.

## Results

### Aim 1: Cross-sectional analyses of protein levels with BMI at baseline

**Figure 1:**
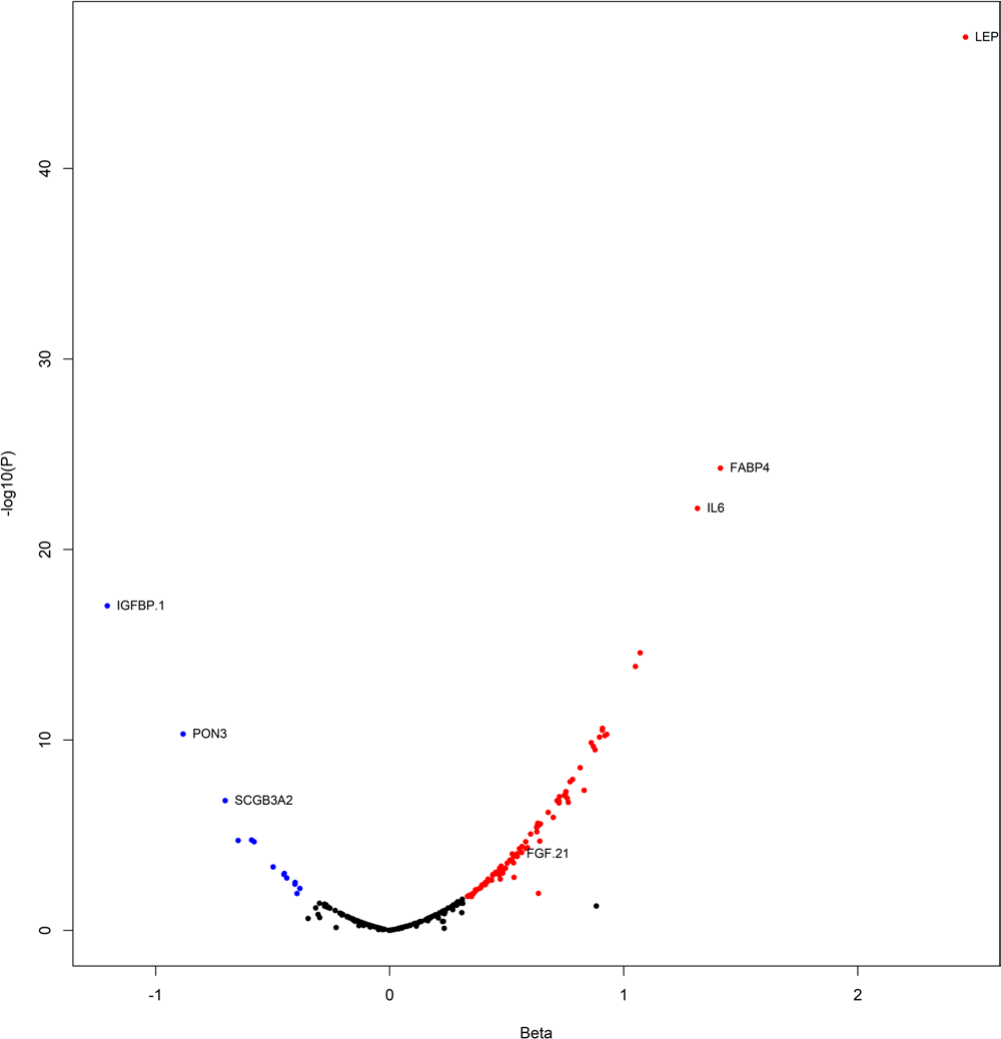
Associations between cross-sectional associations of protein levels and BMI at baseline in DIETFITS. Proteins significantly associated with BMI (at FDR < 5%) are shown in red (positively correlated) or blue (negatively correlated). The top 3 positively and negatively associated proteins are annotated.

The baseline characteristics of the study samples are presented in Table 1. Of 263 tested proteins, we found 102 proteins to be cross-sectionally associated with BMI at baseline (FDR < 5%). Among the 88 positively associated proteins, leptin (LEP, beta=2.46, P = 1.28E-47) and fatty acid binding protein 4 (FABP4, beta=1.41, P = 5.36E-25) were the strongest; while insulin-like growth factor binding protein 1 (IGFBP-1, beta = −1.21, P = 9.12E-18) and paraoxonase 3 (PON3, beta = −0.88, P = 4.93E-11) were the most significant among 14 inversely associated proteins (Fig. 1, Supplementary Table 1). Of these proteins, 80 were measured on CVD II or CVD III, and passed quality control in EpiHealth. Almost all, 75 proteins, were significantly replicated (*P* < 0.05 and in the same direction) for association with BMI among overweight or obese individuals in EpiHealth (Supplementary Table 1).

**Table 1:** Baseline characteristics of DIETFITS and EpiHealth.

	DIETFITS (N = 609)	EpiHealth (N = 1,584)
Age, years	39.8 (6.8)	60.2 (8.3)
Female sex	345 (56.7)	700 (44.2)
Ethnicity		
White	358 (58.8)	1584 (100)
Hispanic	128 (21.0)	0 (0)
Asian	60 (9.9)	0 (0)
African American	23 (3.8)	0 (0)
Am Indian/Alaskan/Pacific Islander	3 (0.5)	0 (0)
Other	37 (6.0)	0 (0)
Weight, kg	96.9 (15.2)	85.1 (12.6)
BMI, kg/m^2^	33.4 (3.3)	28.5 (3.1)
Body fat percentage by DXA	36.4 (6.7)	32.4 (7.9)
HDL, mmol/L	1.3 (0.2)	1.4 (0.4)
LDL, mmol/L	2.9 (0.7)	3.9 (1.0)
TG, mmol/L	1.4 (0.9)	1.4 (0.8)
Total cholesterol, mmol/L	4.9 (0.9)	5.9 (1.1)
Systolic blood pressure, mmHg	123 (12)	137 (16)
Diastolic blood pressure, mmHg	81 (8)	85 (9)
Current smoking	0 (0)	105 (6.6)
Lipid medication	0 (0)	202 (12.8)

Data are presented as mean (standard deviation) or N (%).

### Aim 2: Longitudinal changes in protein levels and BMI during 6 months

**Figure 2:**
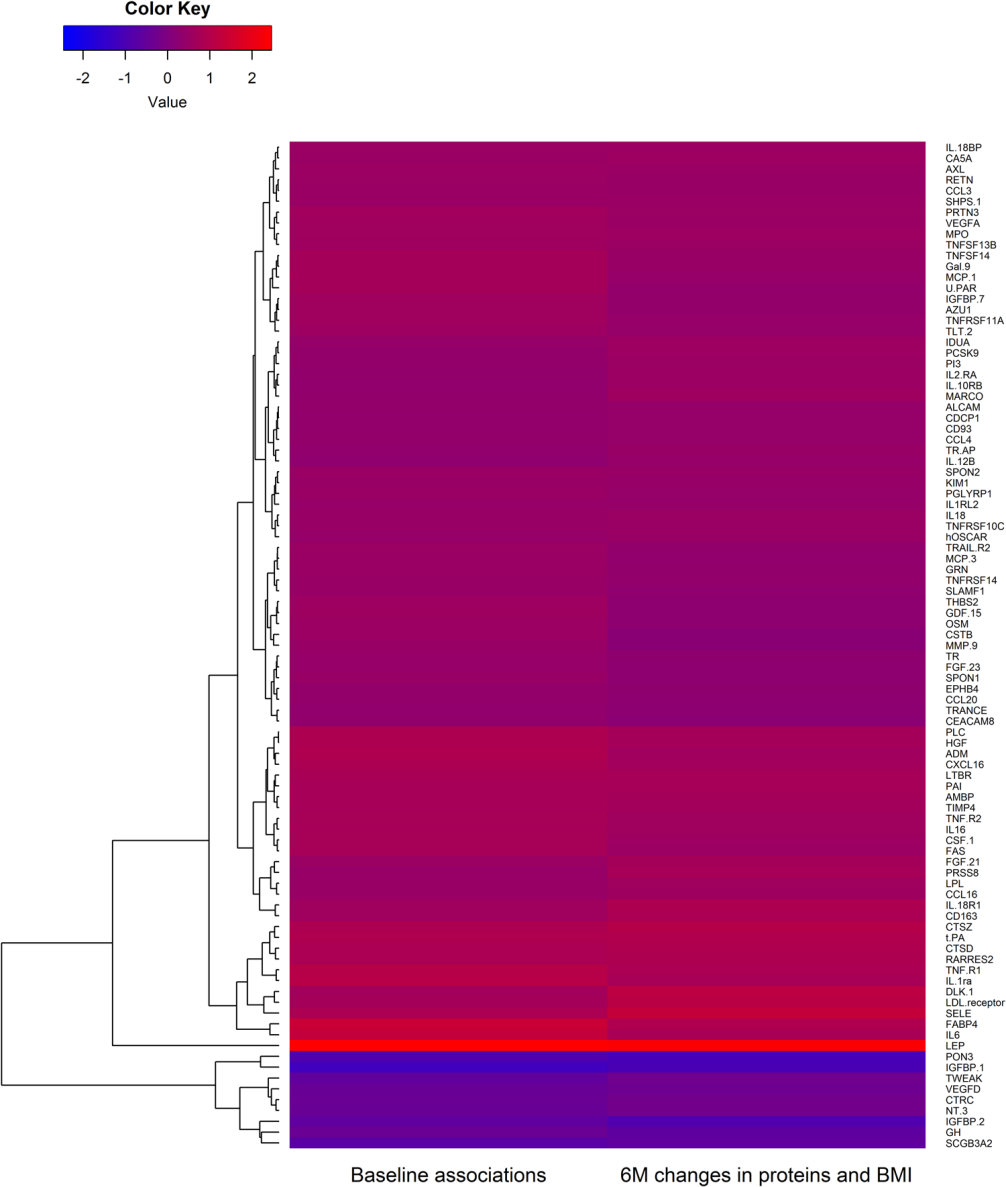
Cross-sectional associations between proteins and BMI at baseline (left panel); and associations between changes in proteins and changes in BMI during 6 months (right panel). Results are shown for 93 proteins significantly associated in both analyses. Red colors indicate positive coefficients (of baseline levels or changes, respectively), while blue colors indicate negative coefficients.

Of 263 tested proteins, 130 were significantly associated with changes in BMI (Supplementary Table 2). Of these, 118 tracked with BMI (i.e. decreased protein levels upon weight loss; positive beta coefficients), while 12 displayed opposite directions (i.e. increased protein levels upon weight loss; negative beta coefficients). Individuals who showed the largest BMI decrease (represented by the 75th percentile [dashed line] in Supplementary Fig. 2) demonstrated the largest decrease (steepest negative slope) in leptin, FABP4 and IL-6; and the largest increase (steepest positive slope) in PON3, IGFBP-1 and SCGB3A2. The 93 proteins that were significantly associated with baseline BMI and additionally significantly associated with changes in BMI are presented in Fig. 2, where positive associations are depicted in red and negative in blue.

### Aim 3: Prediction of weight loss success using circulating proteins at baseline

The mean weight reduction after 3 months of dietary intervention was 5.4 kg (standard deviation [SD], 4.1 kg) on HLF and 6.5 kg (SD, 4.5 kg) on HLC (P = 0.004). There was no significant difference in weight change between a HLF diet vs a HLC diet in the primary analysis of the weight loss intervention as reported previously, where weight loss during the whole 12-month period was the prespecified main outcome^17^. The mean ΔBMI was −2.1 kg/m^2^ (SD, 1.4; range −6.1 to 6.2). Only fibroblast growth factor 21 (FGF-21) significantly predicted weight loss when adjusted for potential confounders (age, sex and race) and correcting for multiple testing (beta = −0.28, P = 7.22E-06, Supplementary Table 3). The negative beta in this analysis should be interpreted such that one SD higher baseline FGF-21 levels were associated with 0.28 kg/m^2^ lower BMI during the first three months of dietary intervention. There were no significant interactions between baseline protein levels and diet type on weight loss (Supplementary Table 4**)**.

## Discussion

### Principal findings

In the present study, we analyzed 263 inflammatory and cardiovascular proteins before and after weight loss in a randomized dietary interventional trial of 609 overweight/obese individuals. Our main results were four-fold: (1) We found 102 proteins to be associated with BMI at baseline; (2) In longitudinal analyses, 130 proteins tracked with weight loss; (3) Baseline levels of FGF-21 could significantly predict weight loss; and (4) There were no interactions between baseline protein levels and diet type on weight loss. Hence, our overall results indicate that studies of circulating proteins in relation to obesity and weight loss can be informative regarding the pathophysiology of obesity, while they have limited value for prediction of weight loss and individualization of dietary assignments.

### Associations of proteins with obesity and weight loss

Our findings include some positive controls, such as the expected associations of leptin^25^ and FABP4^26^ with BMI and weight loss. Indeed, the decreases in leptin levels during weight loss that we observed are consistent with a recent study that studied –omics profiles of 23 overweight/obese individuals during a short (60 day) weight loss intervention^5^. Other studies investigated proteomic profiles of overweight and obese individuals who underwent weight loss via caloric restriction (800 kcal/day for 8 weeks)^9,12,15^; thus, significantly different in design from DIETFITS. The differences also include proteomics via mass spectrometry in these prior studies as compared to our study using affinity-based proteomics. However, most of our findings regarding associations of circulating proteins with BMI and weight loss are novel. One such example was our observation that circulating proprotein convertase subtilisin/kexin type 9 (PCSK9) levels were positively correlated with BMI, as well as tracked with weight loss. This extends prior smaller studies where PCSK9 has been positively associated with obesity cross-sectionally^27,28^, and is particularly notable in light of the known role of PCSK9 levels in LDL cholesterol metabolism and atherosclerosis development^29–31^. Another interesting observation is that elevated AXL Receptor Tyrosine Kinase (AXL) levels among obese individuals decreased upon weight loss. AXL is aberrantly overexpressed in a variety of malignancies^32^, and activation of AXL is strongly linked to cell proliferation, survival, migration, and invasion by activating the oncogenic signaling pathways, including PI3K/Akt and/or MAPK/Erk pathways. Recently, AXL inhibitors have been proposed as novel cancer therapeutic agents^32^, thus in this light the link between decreasing AXL upon weight loss is worth further investigation to uncover potential biological mechanism. Further, we observed several proteins that were negatively associated with baseline BMI and also increased with weight loss. These include insulin-like growth factor-binding protein 2 (IGFBP2), which is in agreement with a previous observation of increases of IGFBP2 after surgical weight loss, and inversely associated with incident metabolic syndrome and diabetes mellitus, after adjusting for established cardiometabolic disease risk factors in the general population^11^. Other proteins showing similar pattern of inverse associations with BMI in the cross-sectional and longitudinal analyses included IGFBP-1, PON3, SCGB3A2, TWEAK, NT-3, VEGF-D, CTRC and GH.

In previous study, after 8-week weight loss during a low caloric diet, proteins of the acute phase response indicated a reduction in low-grade inflammation since C-reactive protein, alpha-1-acid glycoprotein, alpha-1 antitrypsin and serum amyloid A levels dropped^15^. However, none of the reported proteins was analyzed in the present study, thus no comparison could be performed. Only adipokine leptin was consistently associated with BMI change in our and previous diet-induced weight loss studies^5,9,14^. We found similar protein biomarkers that tracked changes in BMI in our study and were also altered after surgical weight loss and those include CD163, SELE, PAI-1, tPA, IGFBP1, IGFBP2, SELP, COL1A1, ITGB2, LDL receptor, PON3, CTSD^11^.

### Weight loss prediction and personalized dietary advice

Obesity has become a worldwide public health epidemic, and dietary intervention – often coupled with increased exercise – is the most common approach to weight loss among obese and overweight individuals. Since reducing weight is of such tremendous importance from a health perspective and yet halting and reversing the obesity epidemic has proven to be so daunting, it is critical to better understand factors predicting the chance for successful weight loss^33^. In the era of precision medicine, there is an increasing interest in novel biomarkers, and the extended list of potentially promising biomarkers, such as adipokines, cytokines, metabolites, and microRNAs, implicated in obesity may bring promise for improved, personalized prevention^4^. For example, there is an invention that provides gender-specific biomarkers (sex hormone-binding globulin paraoxonase/arylesterase 1 and beta-2-microglobulin) that allow prediction of weight trajectory of male subject prior to a low calorie diet^34^. Another invention utilizes serum concentration of hormones for predicting weight loss success for a person undergoing gastric banding^35^. In the present study, after correction for multiple testing, baseline FGF-21 was the only protein that significantly predicted weight loss. FGF-21 is a potent metabolic regulator shown to improve glucose and lipid metabolism^36–38^, as well as to reduce overall body weight and adipose mass in rodents^39^. Moreover, FGF-21 is an independent predictor of the metabolic syndrome and diabetes in healthy Caucasians^40^ and correlates positively with obesity where paradoxical increase of serum FGF-21 in obese individuals may be explained by a compensatory response or resistance to FGF-21^41^. Genetic variant in *FGF21* has been associated with dietary macronutrient intake in a genome-wide meta-analysis of 33,355 subjects^42^ and FGF-21 mediates endocrine control of simple sugar intake and sweet taste preference by the liver in mice^43^. Thus, our results highlight the potential of FGF-21 levels in identifying subjects more prone to weight loss. In contrast, we did not observe any significant interactions between blood protein levels at baseline and diet type on weight loss. Hence, our results suggest that the set of plasma protein biomarkers assessed in these analyses have limited utility for personalized dietary advice.

### Strength and limitations

The main strength of this study is the large number study samples, i.e. 609 healthy obese individuals, compared to previous studies that investigated proteomics in relation to diet and weight loss performed in animal models^44^ or included small human samples^5,8^. Another strength is the large number of measured proteins and the repeated measurements, i.e. baseline and after 6 months of diet, which allowed us to investigate individual changes in those protein levels. Moreover, we replicated results from the cross-sectional analyses in overweight/obese subjects from a cohort study from the general population. There are also some limitations to our study. Since we used a target proteomic chip designed with proteins linked to cardiovascular disease or inflammation, we cannot exclude possibility that there are other proteins with better predictive potential for weight loss. Moreover, there is no other study designed in a similar way to serve as a replication cohort to further confirm the FGF-21 finding. However, we corrected the analysis for false positives, as well as there are several previous studies linking FGF-21 with obesity and diet^39,41–43^, thus we believe the association observed in our study is not a random finding. Finally, the generalizability of our findings to other populations is unknown.

## Conclusions

In conclusion, many of the examined inflammatory and cardiovascular biomarkers were associated with body mass index and tracked with weight loss shedding light into the pathophysiology of obesity. However, out of 263 proteins analyzed at baseline, only FGF-21 predicted weight loss, and none helped individualize dietary assignment.

## Supplementary information

Supplementary Information.
