# Supplementary material for: Proteomic profiles before and during weight loss: Results from randomized trial of dietary intervention
Source: Sci Rep. 2020 May 13;10:7913. doi: 10.1038/s41598-020-64636-7 (PMC7220904; doi:10.1038/s41598-020-64636-7)
Supplement: Supplementary file 1 — Supplementary Information. [file 41598_2020_64636_MOESM1_ESM.pdf]

<u>Supplementary Information</u>

Proteomic profiles before and during weight loss: Results from randomized trial of dietary intervention.

Sylwia M. Figarska, Joseph Rigdon, Andrea Ganna, Sölve Elmståhl, Lars Lind, Christopher D. Gardner, Erik Ingelsson

Supplementary Figure 1. Aims of the study and summary of main results.

Supplementary Figure 2. Associations of changes in BMI and changes in protein levels for the three most significant positive and negative associations.

Supplementary Table 1. Associations between blood protein levels and BMI at baseline (linear regression analyses adjusted for age, sex and race) in DIETFITS and EpiHealth.

Supplementary Table 2. Associations between changes in blood proteins and changes in BMI during 6 months.

Supplementary Table 3. Associations between blood protein levels at baseline and weight loss ($\Delta BMI = BMI_{3months} - BMI_{baseline}$). Data are from linear regression analyses adjusted for age, sex and race.

Supplementary Table 4. Interaction effect of blood protein levels at baseline and diet on weight loss ($\Delta BMI = BMI_{3months} - BMI_{baseline}$). Data are from linear regression analyses including blood protein*diet interaction term, adjusted for age, sex and race.

Supplementary Figure 1. Aims of the study and main results.

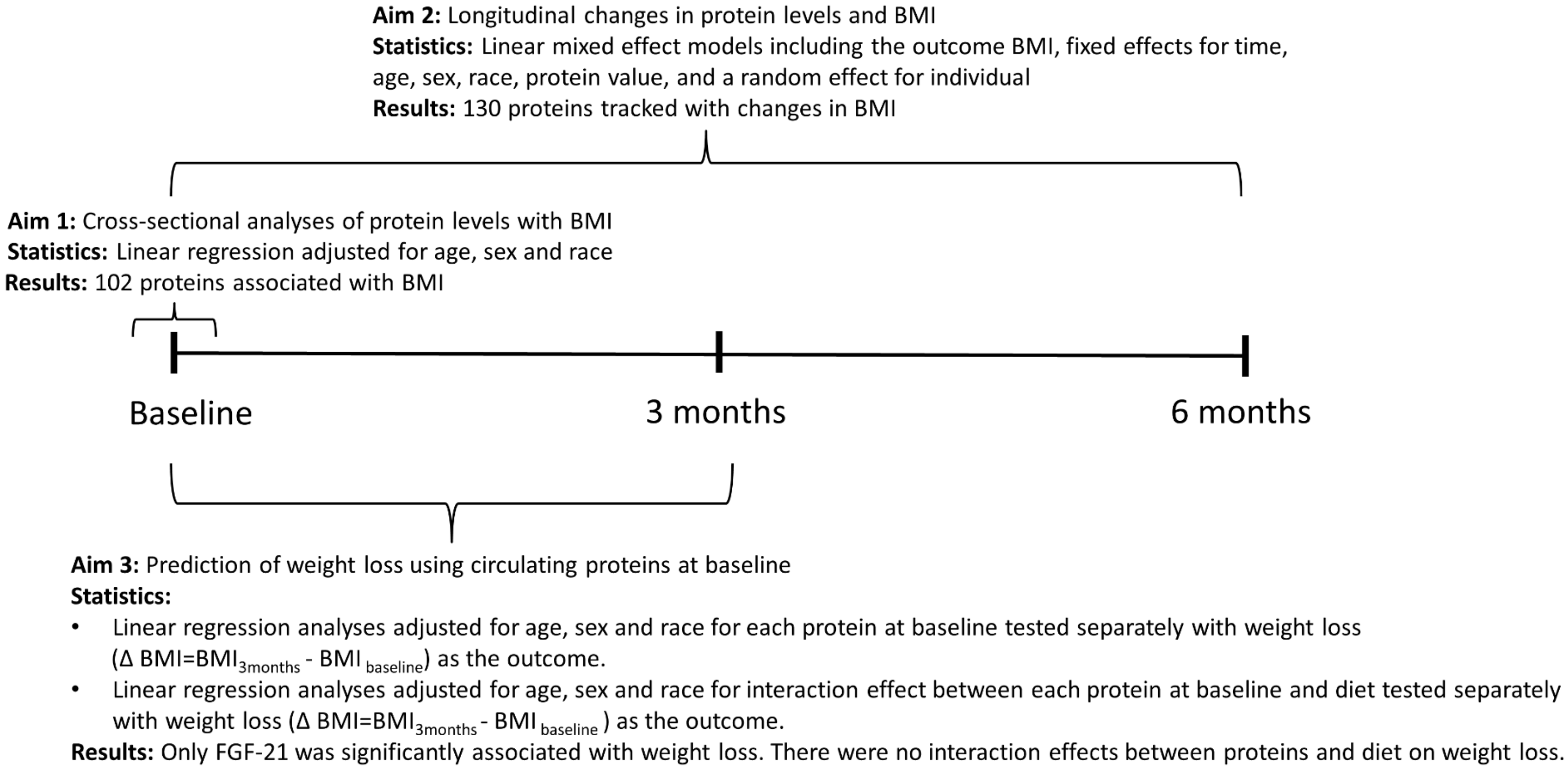

Supplementary Figure 2. **Associations of changes in BMI and changes in blood protein levels for the three most significant positive and negative associations.** (A) Leptin (LEP), (B) Fatty acid binding protein 4 (FABP4), (C) Interleukin 6 (IL-6), (D) Insulin-like growth factor-binding protein 1 (IGFBP-1), (E) Paraoxonase (PON3) and (F) Secretoglobin family 3A member 2 (SCGB3A2). The top 3 positive and negative strongest associations in Aim1. The graphs show the changes over time of protein normalized values for the 25th, 50th and 75th percentile of weight loss. The value of the protein at baseline is set to the median for all three groups of change in BMI to help interpretation of the graphs.

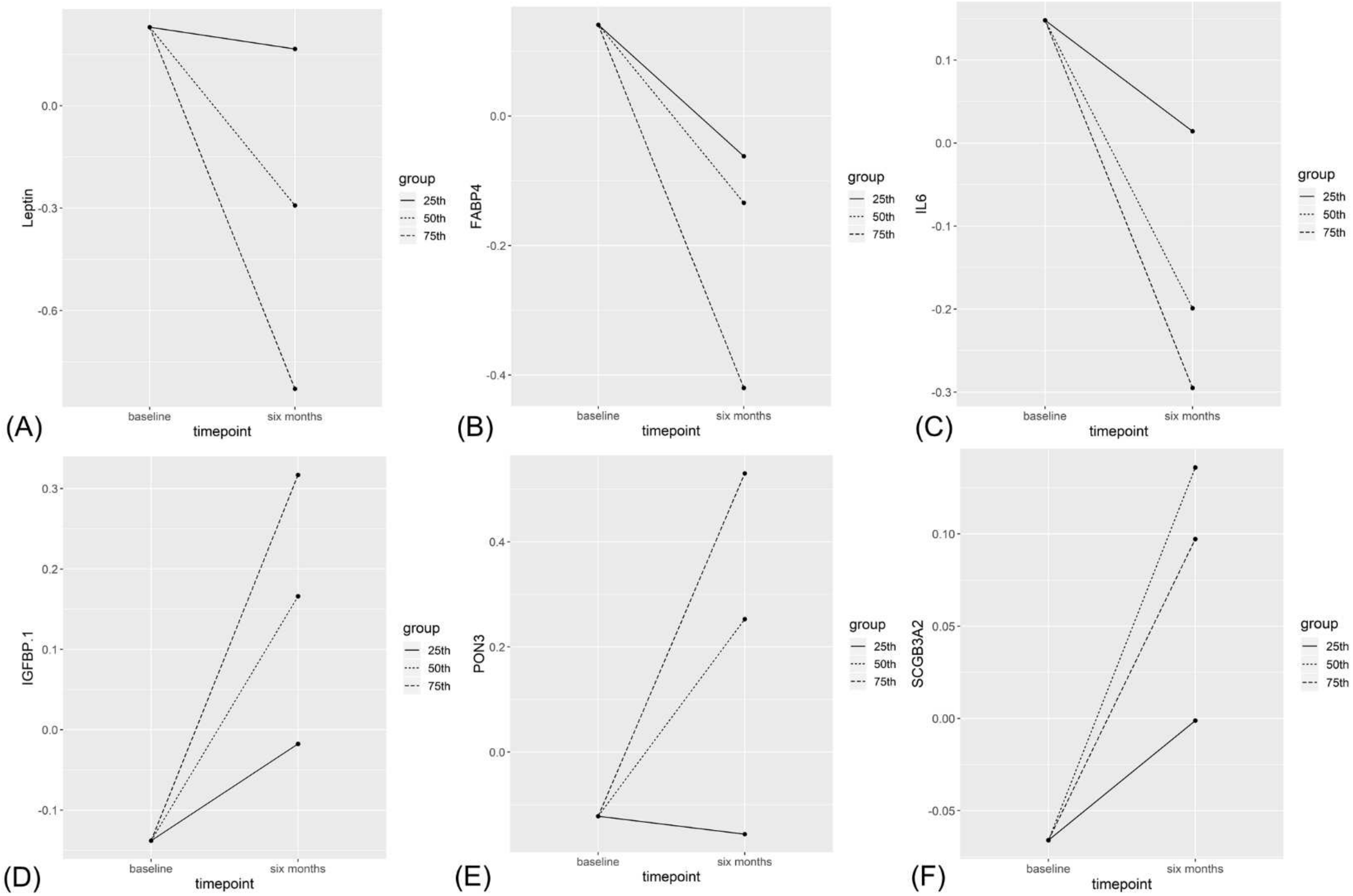

Supplementary Table 1. Associations between blood protein levels and BMI at baseline (linear regression analyses adjusted for age, sex and race) in DIETFITS and EpiHealth. Significant P-values in DIETFITS (FDR of 5%) and in EpiHealth (P<0.05) are in bold.
[a]Not available in EpiHealth
[b]No replication attempt since associations were non-significant in DIETFITS

**Protein**	**DIETFITS**			**EpiHealth**		
	Beta	SE	P	Beta	SE	P
Leptin (LEP)	2.461	0.155	**1.28E-47**	2.467	0.090	**4.67E-135**
Fatty acid-binding protein, adipocyte (FABP4)	1.413	0.131	**5.36E-25**	1.767	0.082	**3.34E-91**
Interleukin-6 (IL-6)	1.315	0.128	**6.89E-23**	1.093	0.079	**6.14E-41**
Insulin-like growth factor-binding protein 1 (IGFBP-1)	-1.207	0.136	**9.12E-18**	-1.047	0.077	**3.89E-40**
Interleukin-1 receptor antagonist protein (IL-1ra)	1.070	0.132	**2.66E-15**	1.346	0.076	**5.39E-63**
Tumor necrosis factor receptor 1 (TNF-R1)	1.050	0.133	**1.37E-14**	0.762	0.080	**5.78E-21**
Perlecan (PLC)	0.910	0.134	**2.45E-11**	0.732	0.080	**1.23E-19**
Hepatocyte growth factor (HGF)	0.909	0.134	**3.08E-11**	a		
Paraoxonase (PON3)	-0.883	0.132	**4.93E-11**	-1.090	0.076	**1.88E-43**
Tissue-type plasminogen activator (t-PA)	0.928	0.139	**5.03E-11**	0.851	0.079	**2.00E-26**
ADM (ADM)	0.919	0.138	**5.93E-11**	1.147	0.084	**1.57E-40**
Cathepsin Z (CTSZ)	0.897	0.135	**7.11E-11**	0.647	0.079	**4.25E-16**
Retinoic acid receptor responder protein 2 (RARRES2)	0.861	0.132	**1.39E-10**	1.070	0.077	**4.93E-41**
C-X-C motif chemokine 16 (CXCL16)	0.870	0.135	**2.18E-10**	0.287	0.080	**3.33E-04**
E-selectin (SELE)	0.878	0.137	**3.28E-10**	0.720	0.077	**1.91E-20**
Lymphotoxin-beta receptor (LTBR)	0.814	0.135	**2.85E-09**	0.375	0.080	**2.65E-06**
Macrophage colony-stimulating factor 1 (CSF-1)	0.781	0.135	**1.18E-08**	a		
Tumor necrosis factor receptor 2 (TNF-R2)	0.771	0.134	**1.57E-08**	0.542	0.080	**2.17E-11**
Cathepsin D (CTSD)	0.831	0.150	**4.42E-08**	0.793	0.082	**1.28E-21**
Plasminogen activator inhibitor 1 (PAI)	0.753	0.137	**5.12E-08**	0.778	0.076	**8.30E-24**
Protein AMBP (AMBP)	0.751	0.137	**6.78E-08**	0.265	0.082	**1.22E-03**
Pro-interleukin-16 (IL16)	0.746	0.137	**8.20E-08**	0.491	0.081	**1.34E-09**
Tumor necrosis factor ligand superfamily member 14 (TNFSF14)	0.726	0.134	**9.54E-08**	a		
Metalloproteinase inhibitor 4 (TIMP4)	0.758	0.141	**1.16E-07**	0.465	0.086	**7.01E-08**

Protein	DIETFITS			EpiHealth		
Monocyte chemotactic protein 1 (MCP-1)	0.715	0.135	**1.54E-07**	0.425	0.081	**1.64E-07**
Secretoglobin family 3A member 2 (SCGB3A2)	-0.703	0.132	**1.55E-07**	-0.604	0.079	**3.49E-14**
Galectin-9 (Gal-9)	0.723	0.136	**1.62E-07**	0.767	0.084	**2.48E-19**
Low-density lipoprotein receptor (LDL receptor)	0.718	0.135	**1.62E-07**	0.608	0.077	**4.78E-15**
Tumor necrosis factor receptor superfamily member 6 (FAS)	0.763	0.145	**1.89E-07**	0.516	0.080	**1.59E-10**
Urokinase plasminogen activator surface receptor (U-PAR)	0.724	0.138	**2.05E-07**	0.645	0.084	**2.96E-14**
Myeloblastin (PRTN3)	0.677	0.134	**6.30E-07**	0.295	0.078	**1.72E-04**
Protein delta homolog 1 (DLK-1)	0.699	0.142	**1.17E-06**	0.266	0.080	**8.36E-04**
Scavenger receptor cysteine-rich type 1 protein M130 (CD163)	0.633	0.133	**2.34E-06**	0.645	0.078	**3.51E-16**
Vascular endothelial growth factor A (VEGF-A)	0.644	0.136	**2.57E-06**	a		
Tumor necrosis factor receptor superfamily member 11A (TNFRSF11A)	0.636	0.135	**3.10E-06**	0.751	0.082	**1.28E-19**
Myeloperoxidase (MPO)	0.630	0.134	**3.22E-06**	0.259	0.079	**1.09E-03**
Azurocidin (AZU1)	0.628	0.135	**3.91E-06**	0.388	0.078	**7.93E-07**
Interleukin-18 receptor 1 (IL-18R1)	0.629	0.138	**6.58E-06**	a		
Tumor necrosis factor ligand superfamily member 13B (TNFSF13B)	0.602	0.134	**8.74E-06**	0.306	0.083	**2.19E-04**
Insulin-like growth factor-binding protein 2 (IGFBP-2)	-0.590	0.136	**1.79E-05**	-0.981	0.078	**1.92E-34**
Delta and Notch-like epidermal growth factor-related receptor (DNER)	-0.647	0.150	**1.92E-05**	a		
Insulin-like growth factor-binding protein 7 (IGFBP-7)	0.641	0.149	**2.03E-05**	0.454	0.080	**1.98E-08**
Tumor necrosis factor (Ligand) superfamily, member 12 (TWEAK)	-0.579	0.135	**2.20E-05**	NA	NA	**NA**
Thrombospondin-2 (THBS2)	0.581	0.136	**2.22E-05**	0.526	0.079	**4.44E-11**
Cystatin-B (CSTB)	0.565	0.136	**3.92E-05**	0.910	0.080	**5.37E-29**
Growth/differentiation factor 15 (GDF-15)	0.589	0.143	**4.47E-05**	0.679	0.094	**6.89E-13**
Trem-like transcript 2 protein (TLT-2)	0.581	0.142	**4.95E-05**	0.326	0.079	**3.45E-05**
Oncostatin-M (OSM)	0.554	0.136	**5.19E-05**	a		
Tyrosine-protein kinase receptor UFO (AXL)	0.564	0.142	**8.21E-05**	0.143	0.078	6.73E-02
Fibroblast growth factor 21 (FGF-21)	0.544	0.138	**9.53E-05**	0.698	0.079	**2.35E-18**
Granulins (GRN)	0.524	0.134	**9.64E-05**	0.227	0.079	**4.23E-03**
TNF-related apoptosis-inducing ligand receptor 2 (TRAIL-R2)	0.543	0.139	**1.06E-04**	0.729	0.087	**1.06E-16**
Resistin (RETN)	0.539	0.139	**1.18E-04**	0.391	0.079	**7.22E-07**

Protein	DIETFITS			EpiHealth		
C-C motif chemokine 3 (CCL3)	0.529	0.137	**1.30E-04**	0.669	0.081	**3.54E-16**
Interleukin-18-binding protein (IL-18BP)	0.545	0.142	**1.32E-04**	0.337	0.081	**3.55E-05**
Tyrosine-protein phosphatase non-receptor type substrate 1 (SHPS-1)	0.514	0.138	**2.05E-04**	0.217	0.079	**6.24E-03**
Peptidoglycan recognition protein 1 (PGLYRP1)	0.524	0.142	**2.35E-04**	0.358	0.079	**5.85E-06**
Carbonic anhydrase 5A, mitochondrial (CA5A)	0.529	0.145	**2.81E-04**	a		
Spondin-2 (SPON2)	0.502	0.138	**2.91E-04**	0.522	0.082	**2.12E-10**
Matrix metalloproteinase-9 (MMP-9)	0.477	0.134	**4.18E-04**	0.610	0.078	**1.12E-14**
C-C motif chemokine 28 (CCL28)	-0.498	0.142	**4.64E-04**	a		
C-C motif chemokine 16 (CCL16)	0.495	0.142	**5.43E-04**	0.758	0.079	**3.95E-21**
Tumor necrosis factor receptor superfamily member 14 (TNFRSF14)	0.470	0.135	**5.46E-04**	0.453	0.081	**2.52E-08**
Spondin-1 (SPON1)	0.477	0.138	**5.77E-04**	0.203	0.084	**1.64E-02**
Interleukin-18 (IL-18)	0.487	0.142	**6.17E-04**	0.346	0.082	**2.63E-05**
Tumor necrosis factor receptor superfamily member 10C (TNFRSF10C)	0.469	0.137	**6.32E-04**	0.369	0.078	**2.27E-06**
Signaling lymphocytic activation molecule (SLAMF1)	0.470	0.139	**7.82E-04**	a		
Transferrin receptor protein 1 (TR)	0.453	0.135	**8.88E-04**	0.423	0.078	**6.78E-08**
Kidney injury molecule 1 (KIM-1)	0.483	0.146	**9.85E-04**	0.474	0.085	**3.36E-08**
Neurotrophin-3 (NT-3)	-0.450	0.136	**1.01E-03**	a		
Interleukin-1 receptor-like 2 (IL1RL2)	0.452	0.137	**1.03E-03**	0.211	0.082	**1.00E-02**
Matrix metalloproteinase-10 (MMP-10)	-0.452	0.138	**1.15E-03**	a		
C-C motif chemokine 22 (CCL22)	0.442	0.135	**1.16E-03**	0.240	0.078	**2.21E-03**
Prostasin (PRSS8)	0.468	0.144	**1.24E-03**	0.681	0.084	**1.46E-15**
Fibroblast growth factor 23 (FGF-23)	0.440	0.136	**1.28E-03**	0.552	0.081	**1.27E-11**
Monocyte chemotactic protein 3 (MCP-3)	0.531	0.168	**1.64E-03**	a		
Vascular endothelial growth factor D (VEGF-D)	-0.440	0.140	**1.76E-03**	-0.421	0.081	**2.11E-07**
Alpha-L-iduronidase (IDUA)	0.420	0.135	**2.02E-03**	0.335	0.080	**3.17E-05**
Lipoprotein lipase (LPL)	0.472	0.152	**2.03E-03**	-0.295	0.090	**1.03E-03**
Osteoclast-associated immunoglobulin-like receptor (hOSCAR)	0.436	0.142	**2.32E-03**	0.125	0.082	1.29E-01
Ephrin type-B receptor 4 (EPHB4)	0.418	0.139	**2.76E-03**	0.133	0.079	9.44E-02
Kallikrein-6 (KLK6)	-0.404	0.136	**3.00E-03**	-0.347	0.078	**9.84E-06**

Protein	DIETFITS			EpiHealth		
Proprotein convertase subtilisin/kexin type 9 (PCSK9)	0.409	0.137	**3.03E-03**	0.278	0.081	**6.32E-04**
Chymotrypsin C (CTRC)	-0.405	0.139	**3.73E-03**	-0.218	0.080	**6.53E-03**
C-C motif chemokine 4 (CCL4)	0.409	0.141	**3.94E-03**	a		
Osteopontin (OPN)	0.395	0.137	**4.04E-03**	0.207	0.080	**1.01E-02**
C-C motif chemokine 20 (CCL20)	0.393	0.138	**4.64E-03**	a		
Elafin (PI3)	0.391	0.139	**5.11E-03**	0.300	0.080	**1.99E-04**
CD166 antigen (ALCAM)	0.390	0.140	**5.36E-03**	0.221	0.083	**8.05E-03**
CUB domain-containing protein 1 (CDCP1)	0.386	0.140	**6.19E-03**	a		
Receptor for advanced glycosylation end products (RAGE)	-0.384	0.140	**6.26E-03**	-0.441	0.080	**3.94E-08**
Complement component C1q receptor (CD93)	0.374	0.138	**7.07E-03**	0.003	0.079	9.71E-01
Macrophage receptor MARCO (MARCO)	0.366	0.136	**7.37E-03**	0.249	0.082	**2.34E-03**
Interleukin-2 receptor subunit alpha (IL2-RA)	0.365	0.139	**9.01E-03**	0.379	0.079	**1.90E-06**
Leukemia inhibitory factor (LIF)	0.635	0.250	**1.12E-02**	a		
Growth hormone (GH)	-0.396	0.156	**1.15E-02**	-0.527	0.089	**3.65E-09**
Carcinoembryonic antigenrelated cell adhesion molecule 8 (CEACAM8)	0.358	0.142	**1.19E-02**	0.489	0.081	**1.97E-09**
TNF-related activation-induced cytokine (TRANCE)	0.351	0.140	**1.22E-02**	a		
Protein S100-A12 (EN-RAGE)	0.337	0.138	**1.47E-02**	a		
Interleukin-10 receptor subunit beta (IL-10RB)	0.341	0.141	**1.60E-02**	a		
Interleukin-12 subunit beta (IL-12B)	0.332	0.138	**1.64E-02**	a		
Tartrate-resistant acid phosphatase type 5 (TR-AP)	0.350	0.146	**1.65E-02**	0.137	0.080	8.70E-02
Lectin-like oxidized LDL receptor 1 (LOX-1)	0.310	0.136	2.28E-02			
Integrin beta-2 (ITGB2)	0.310	0.137	2.38E-02			
Galectin-3 (Gal-3)	0.303	0.140	3.02E-02			
von Willebrand factor (vWF)	0.291	0.135	3.14E-02			
Low affinity immunoglobulin gamma Fc region receptor II-b (IgG Fc receptor II-b)	0.301	0.140	3.15E-02	b		
Placenta growth factor (PlGF)	0.301	0.144	3.68E-02			
Hydroxyacid oxidase 1 (HAOX1)	0.312	0.150	3.79E-02			
Growth/differentiation factor 2 (GDF-2)	-0.300	0.144	3.79E-02			
Transforming growth factor alpha (TGF-alpha)	0.281	0.137	4.01E-02			

Protein	DIETFITS			EpiHealth
CD40L receptor (CD40)	-0.279	0.136	4.14E-02	
Epithelial cell adhesion molecule (Ep-CAM)	-0.272	0.136	4.49E-02	
C-X-C motif chemokine 10 (CXCL10)	0.272	0.137	4.70E-02	
Prolargin (PRELP)	0.287	0.144	4.74E-02	
TNF-related apoptosis-inducing ligand (TRAIL)	0.286	0.144	4.78E-02	
Cathepsin L1 (CTSL1)	0.276	0.139	4.84E-02	
Cystatin D (CST5)	-0.276	0.140	4.88E-02	
Interleukin-1 alpha (IL-1 alpha)	0.883	0.454	5.24E-02	
Gastrotropin (GT)	-0.278	0.144	5.33E-02	
Gastric intrinsic factor (GIF)	-0.268	0.140	5.51E-02	
Interleukin-6 receptor subunit alpha (IL-6RA)	0.265	0.138	5.55E-02	
T-cell surface glycoprotein CD4 (CD4)	0.261	0.139	6.12E-02	
Tissue factor (TF)	-0.266	0.142	6.12E-02	
Stem cell factor (SCF)	-0.262	0.140	6.13E-02	
Matrix metalloproteise-3 (MMP-3)	-0.316	0.172	6.60E-02	
Intercellular adhesion molecule 2 (ICAM-2)	0.250	0.136	6.70E-02	
Carboxypeptidase A1 (CPA1)	-0.255	0.140	6.80E-02	
ST2 protein (ST2)	0.270	0.154	8.12E-02	
Follistatin (FS)	0.237	0.138	8.73E-02	
CD40 ligand (CD40-L)	-0.233	0.136	8.86E-02	
Cadherin-5 (CDH5)	0.228	0.136	9.38E-02	
Brain-derived neurotrophic factor (BDNF)	0.237	0.146	1.06E-01	
Matrix metalloproteise-2 (MMP-2)	0.222	0.138	1.08E-01	
Glial cell line-derived neurotrophic factor (GDNF)	0.236	0.147	1.09E-01	
Neurogenic locus notch homolog protein 3 (Notch 3)	0.225	0.141	1.12E-01	
Poly [ADP-ribose] polymerase 1 (PARP-1)	0.224	0.141	1.12E-01	
Interleukin-1 receptor type 2 (IL-1RT2)	0.223	0.142	1.16E-01	
Interleukin-5 (IL-5)	0.308	0.196	1.16E-01	
C-C motif chemokine 19 (CCL19)	0.214	0.137	1.20E-01	
Fms-related tyrosine kise 3 ligand (Flt3L)	0.223	0.143	1.20E-01	

Protein	DIETFITS			EpiHealth
Serine protease 27 (PRSS27)	-0.213	0.138	1.23E-01	
Proto-oncogene tyrosine-protein kise Src (SRC)	-0.206	0.136	1.30E-01	
Angiotensin-converting enzyme 2 (ACE2)	0.234	0.156	1.34E-01	
Brother of CDO (Protein BOC)	0.209	0.140	1.34E-01	
Artemin (ARTN)	-0.306	0.209	1.43E-01	
Axin-1 (AXIN1)	-0.200	0.138	1.48E-01	
Interleukin-17D (IL-17D)	-0.204	0.141	1.50E-01	
Aminopeptidase N (AP-N)	0.206	0.143	1.50E-01	
Galectin-4 (Gal-4)	0.196	0.136	1.50E-01	
Decorin (DCN)	-0.204	0.144	1.56E-01	
Tumor necrosis factor receptor superfamily member 10A (TNFRSF10A)	0.193	0.137	1.61E-01	
TNF-beta (TNFB)	0.190	0.138	1.69E-01	
Tissue factor pathway inhibitor (TFPI)	0.198	0.145	1.71E-01	
Interleukin-4 receptor subunit alpha (IL-4RA)	0.188	0.138	1.72E-01	
Matrix extracellular phosphoglycoprotein (MEPE)	0.201	0.150	1.80E-01	
Junctiol adhesion molecule A (JAM-A)	-0.180	0.136	1.84E-01	
T-cell surface glycoprotein CD5 (CD5)	0.182	0.138	1.86E-01	
C-C motif chemokine 15 (CCL15)	0.179	0.137	1.92E-01	
Superoxide dismutase [Mn], mitochondrial (SOD2)	-0.182	0.140	1.95E-01	
Sulfotransferase 1A1 (ST1A1)	-0.176	0.137	2.00E-01	
Interleukin-20 receptor subunit alpha (IL-20RA)	-0.299	0.240	2.13E-01	
Interleukin-1 receptor type 1 (IL-1RT1)	0.169	0.136	2.14E-01	
Interleukin-17C (IL-17C)	0.208	0.169	2.18E-01	
Serpin A12 (SERPI12)	0.174	0.142	2.23E-01	
Fractalkine (CX3CL1)	-0.170	0.139	2.23E-01	
SLAM family member 5 (CD84)	-0.164	0.138	2.34E-01	
STAM-binding protein (STAMPB)	-0.163	0.137	2.36E-01	
Urokise-type plasminogen activator (uPA)	0.162	0.137	2.37E-01	
Interleukin-2 receptor subunit beta (IL-2RB)	-0.349	0.296	2.39E-01	

Protein	DIETFITS			EpiHealth
P-selectin glycoprotein ligand 1 (PSGL-1)	0.168	0.143	2.43E-01	
Tumor necrosis factor receptor superfamily member 13B (TNFRSF13B)	0.162	0.140	2.47E-01	
Proheparin-binding EGF-like growth factor (HB-EGF)	-0.158	0.137	2.49E-01	
Collagen alpha-1(I) chain (COL1A1)	0.163	0.141	2.49E-01	
Platelet endothelial cell adhesion molecule (PECAM-1)	-0.154	0.136	2.56E-01	
Matrix metalloproteise-7 (MMP-7)	0.156	0.139	2.63E-01	
Interleukin-15 receptor subunit alpha (IL-15RA)	0.153	0.142	2.79E-01	
Eotaxin-1 (CCL11)	-0.156	0.145	2.82E-01	
Caspase-3 (CASP-3)	-0.139	0.136	3.05E-01	
Myoglobin (MB)	0.163	0.161	3.11E-01	
Carboxypeptidase B (CPB1)	-0.143	0.142	3.14E-01	
Fibroblast growth factor 19 (FGF-19)	-0.138	0.139	3.21E-01	
Pentraxin-related protein PTX3 (PTX3)	-0.135	0.138	3.29E-01	
Pappalysin-1 (PAPPA)	-0.149	0.153	3.30E-01	
2,4-dienoyl-CoA reductase, mitochondrial (DECR1)	-0.132	0.137	3.35E-01	
Osteoprotegerin (OPG)	0.136	0.141	3.36E-01	
Interleukin-24 (IL-24)	0.230	0.239	3.37E-01	
Monocyte chemotactic protein 4 (MCP-4)	0.132	0.138	3.40E-01	
Interleukin-20 (IL-20)	0.226	0.237	3.41E-01	
Chitise-3-like protein 1 (CHI3L1)	0.127	0.137	3.53E-01	
Adenosine Deamise (ADA)	-0.129	0.139	3.56E-01	
Lymphotactin (XCL1)	0.125	0.138	3.65E-01	
Trefoil factor 3 (TFF3)	-0.128	0.141	3.67E-01	
Interleukin-27 (IL-27)	-0.126	0.140	3.70E-01	
NF-kappa-B essential modulator (NEMO)	-0.122	0.138	3.74E-01	
Interleukin-10 (IL-10)	0.124	0.140	3.76E-01	
Pulmory surfactant-associated protein D (PSP-D)	-0.130	0.148	3.79E-01	
Natural killer cell receptor 2B4 (CD244)	-0.120	0.137	3.84E-01	
Thrombomodulin (TM)	0.124	0.143	3.86E-01	

Protein	DIETFITS			EpiHealth
Agouti-related protein (AGRP)	-0.130	0.151	3.92E-01	
C-X-C motif chemokine 9 (CXCL9)	-0.118	0.139	3.95E-01	
Renin (REN)	-0.116	0.139	4.06E-01	
Chitotriosidase-1 (CHIT1)	0.119	0.146	4.17E-01	
Matrix metalloproteise-1 (MMP-1)	0.111	0.137	4.19E-01	
C-X-C motif chemokine 1 (CXCL1)	-0.109	0.138	4.32E-01	
Protein-glutamine gamma-glutamyltransferase 2 (TGM2)	0.103	0.136	4.49E-01	
Programmed cell death 1 ligand 2 (PD-L2)	-0.103	0.137	4.52E-01	
C-X-C motif chemokine 5 (CXCL5)	-0.104	0.139	4.54E-01	
C-X-C motif chemokine 6 (CXCL6)	-0.095	0.138	4.90E-01	
SIR2-like protein 2 (SIRT2)	-0.088	0.137	5.24E-01	
Interleukin-8 (IL-8)	0.087	0.138	5.28E-01	
Melusin (ITGB1BP2)	-0.084	0.138	5.44E-01	
C-C motif chemokine 25 (CCL25)	-0.085	0.140	5.47E-01	
Heme oxygese 1 (HO-1)	0.088	0.146	5.48E-01	
Interleukin-10 receptor subunit alpha (IL-10RA)	-0.112	0.187	5.48E-01	
Polymeric immunoglobulin receptor (PIgR)	-0.084	0.142	5.52E-01	
Leukemia inhibitory factor receptor (LIF-R)	-0.083	0.139	5.52E-01	
Interleukin-13 (IL-13)	-0.132	0.226	5.60E-01	
Heat shock 27 kDa protein (HSP 27)	-0.076	0.137	5.78E-01	
Proteise-activated receptor 1 (PAR-1)	0.074	0.137	5.88E-01	
triuretic peptides B (BNP)	0.114	0.211	5.91E-01	
Programmed cell death 1 ligand 1 (PD-L1)	0.075	0.144	5.99E-01	
A disintegrin and metalloproteise with thrombospondin motifs 13 (ADAM-TS13)	-0.070	0.137	6.07E-01	
Interleukin-7 (IL-7)	0.067	0.137	6.26E-01	
Serine/threonine-protein kise 4 (STK4)	-0.064	0.137	6.39E-01	
Interleukin-4 (IL-4)	-0.083	0.191	6.63E-01	
C-C motif chemokine 17 (CCL17)	-0.056	0.137	6.82E-01	
V-set and immunoglobulin domain-containing protein 2 (VSIG2)	0.056	0.138	6.83E-01	

Protein	DIETFITS			EpiHealth
Platelet-derived growth factor subunit A (PDGF subunit A)	0.055	0.136	6.88E-01	
T cell surface glycoprotein CD6 isoform (CD6)	0.055	0.138	6.91E-01	
Interleukin-17 receptor A (IL-17RA)	-0.054	0.137	6.94E-01	
Interferon gamma (IFN-gamma)	-0.229	0.601	7.04E-01	
Tumor necrosis factor receptor superfamily member 9 (TNFRSF9)	0.052	0.144	7.18E-01	
Epidermal growth factor receptor (EGFR)	0.049	0.136	7.20E-01	
Bone morphogenetic protein 6 (BMP-6)	-0.049	0.138	7.22E-01	
Fatty acid-binding protein, intestil (FABP2)	0.047	0.137	7.31E-01	
Platelet-derived growth factor subunit B (PDGF subunit B)	-0.046	0.137	7.37E-01	
SLAM family member 7 (SLAMF7)	0.054	0.167	7.46E-01	
Caspase-8 (CASP-8)	0.042	0.138	7.63E-01	
Thymic stromal lymphopoietin (TSLP)	0.233	0.790	7.68E-01	
Angiopoietin-1 (ANG-1)	-0.040	0.137	7.72E-01	
Thrombopoietin (THPO)	-0.039	0.137	7.73E-01	
Beta-nerve growth factor (Beta-NGF)	-0.038	0.138	7.80E-01	
Latency-associated peptide transforming growth factor beta-1 (LAP TGF-beta-1)	0.037	0.137	7.86E-01	
Angiopoietin-1 receptor (TIE2)	0.038	0.141	7.89E-01	
Contactin-1 (CNTN1)	-0.035	0.137	8.00E-01	
Sortilin (SORT1)	-0.034	0.137	8.03E-01	
Interleukin-17A (IL-17A)	0.040	0.173	8.15E-01	
Matrix metalloproteise-12 (MMP-12)	0.029	0.136	8.31E-01	
Fibroblast growth factor 5 (FGF-5)	-0.031	0.159	8.43E-01	
Dickkopf-related protein 1 (Dkk-1)	-0.022	0.137	8.72E-01	
C-C motif chemokine 24 (CCL24)	0.019	0.143	8.95E-01	
Interleukin-33 (IL-33)	-0.048	0.368	8.96E-01	
Neurturin (NRTN)	-0.031	0.239	8.98E-01	
Tumor necrosis factor (TNF)	-0.029	0.232	8.99E-01	
C-C motif chemokine 23 (CCL23)	0.017	0.146	9.05E-01	
Tyrosine-protein kise Mer (MERTK)	0.013	0.140	9.28E-01	

Protein	DIETFITS			EpiHealth
Monocyte chemotactic protein 2 (MCP-2)	0.006	0.139	9.65E-01	
P-selectin (SELP)	-0.004	0.136	9.78E-01	
C-X-C motif chemokine 11 (CXCL11)	-0.004	0.138	9.79E-01	
Eukaryotic translation initiation factor 4E-binding protein 1 (4E-BP1)	0.003	0.137	9.84E-01	
Lactoylglutathione lyase (GLO1)	0.002	0.137	9.89E-01	
Bleomycin hydrolase (BLM hydrolase)	0.002	0.142	9.89E-01	

Supplementary Table 2. Associations between changes in blood proteins and changes in BMI during 6 months. Data are from linear mixed effect model analyses adjusted for age, sex, race and time. Beta coefficients represent SD change in protein level per 1 SD increase in BMI.

**Protein**	**Beta**	**Se**	**P**
Leptin (LEP)	2.384	0.091	**6.53E-111**
E-selectin (SELE)	1.329	0.100	**1.44E-37**
Low-density lipoprotein receptor (LDL receptor)	1.113	0.085	**5.16E-36**
Tissue-type plasminogen activator (t-PA)	0.971	0.076	**4.89E-34**
Cathepsin Z (CTSZ)	1.037	0.085	**7.22E-32**
Protein delta homolog 1 (DLK-1)	1.187	0.101	**8.56E-30**
Paraoxonase (PON3)	-1.089	0.097	**1.14E-27**
Insulin-like growth factor-binding protein 1 (IGFBP-1)	-1.041	0.094	**4.12E-27**
Retinoic acid receptor responder protein 2 (RARRES2)	0.881	0.081	**4.92E-26**
Fatty acid-binding protein, adipocyte (FABP4)	0.924	0.089	**4.64E-24**
Tumor necrosis factor receptor 1 (TNF-R1)	0.833	0.084	**4.08E-22**
Cathepsin D (CTSD)	0.929	0.096	**2.80E-21**
Plasminogen activator inhibitor 1 (PAI)	0.761	0.079	**1.12E-20**
Perlecan (PLC)	0.729	0.076	**1.43E-20**
Fibroblast growth factor 21 (FGF-21)	0.722	0.076	**2.66E-20**
Insulin-like growth factor-binding protein 2 (IGFBP-2)	-0.903	0.096	**3.06E-20**
Interleukin-6 (IL-6)	0.800	0.087	**3.46E-19**
Interleukin-18 receptor 1 (IL-18R1)	0.913	0.102	**1.19E-18**
Scavenger receptor cysteine-rich type 1 protein M130 (CD163)	0.851	0.095	**2.01E-18**
Prostasin (PRSS8)	0.735	0.083	**5.80E-18**
Lymphotoxin-beta receptor (LTBR)	0.751	0.086	**1.33E-17**
Interleukin-1 receptor antagonist protein (IL-1ra)	0.796	0.092	**1.42E-17**
Protein AMBP (AMBP)	0.721	0.085	**6.91E-17**
Hepatocyte growth factor (HGF)	0.726	0.086	**1.76E-16**
Integrin beta-2 (ITGB2)	0.664	0.082	**1.70E-15**
ADM (ADM)	0.646	0.080	**2.49E-15**
Metalloproteinase inhibitor 4 (TIMP4)	0.693	0.088	**9.79E-15**

Protein	Beta	Se	P
C-X-C motif chemokine 16 (CXCL16)	0.681	0.088	**3.13E-14**
Proprotein convertase subtilisin/kexin type 9 (PCSK9)	0.584	0.076	**5.05E-14**
Lipoprotein lipase (LPL)	0.620	0.086	**1.07E-12**
Growth hormone (GH)	-0.632	0.088	**1.29E-12**
Tumor necrosis factor receptor 2 (TNF-R2)	0.635	0.088	**1.36E-12**
Pro-interleukin-16 (IL16)	0.623	0.088	**3.31E-12**
Tumor necrosis factor receptor superfamily member 6 (FAS)	0.553	0.082	**3.59E-11**
Vascular endothelial growth factor A (VEGF-A)	0.533	0.080	**4.69E-11**
Macrophage receptor MARCO (MARCO)	0.606	0.092	**6.96E-11**
Macrophage colony-stimulating factor 1 (CSF-1)	0.570	0.087	**1.20E-10**
Tumor necrosis factor ligand superfamily member 13B (TNFSF13B)	0.550	0.086	**2.41E-10**
Alpha-L-iduronidase (IDUA)	0.575	0.092	**5.49E-10**
Carbonic anhydrase 5A, mitochondrial (CA5A)	0.573	0.093	**1.23E-09**
Myeloperoxidase (MPO)	0.574	0.094	**1.27E-09**
Interleukin-10 receptor subunit beta (IL-10RB)	0.562	0.092	**1.72E-09**
Spondin-2 (SPON2)	0.472	0.077	**1.74E-09**
Interleukin-18-binding protein (IL-18BP)	0.565	0.093	**1.87E-09**
Tumor necrosis factor ligand superfamily member 14 (TNFSF14)	0.484	0.081	**3.34E-09**
C-C motif chemokine 16 (CCL16)	0.594	0.100	**4.66E-09**
Secretoglobin family 3A member 2 (SCGB3A2)	-0.582	0.101	**9.93E-09**
Elafin (PI3)	0.541	0.094	**1.06E-08**
Tyrosine-protein kinase receptor UFO (AXL)	0.526	0.092	**1.22E-08**
Interleukin-2 receptor subunit alpha (IL2-RA)	0.559	0.098	**1.31E-08**
Myeloblastin (PRTN3)	0.542	0.097	**3.07E-08**
Monocyte chemotactic protein 1 (MCP-1)	0.445	0.080	**4.09E-08**
Interleukin-1 receptor type 2 (IL-1RT2)	0.504	0.093	**6.98E-08**
Interleukin-18 (IL-18)	0.539	0.100	**8.36E-08**
C-C motif chemokine 3 (CCL3)	0.478	0.090	**1.33E-07**
Pentraxin-related protein PTX3 (PTX3)	-0.441	0.086	**3.16E-07**
Tartrate-resistant acid phosphatase type 5 (TR-AP)	0.498	0.097	**3.54E-07**

Protein	Beta	Se	P
Azurocidin (AZU1	0.415	0.081	**3.69E-07**
Tumor necrosis factor receptor superfamily member 11A (TNFRSF11A)	0.446	0.087	**3.92E-07**
Galectin-9 (Gal-9)	0.471	0.094	**6.61E-07**
Myoglobin (MB)	0.427	0.085	**6.71E-07**
CD166 antigen (ALCAM)	0.423	0.084	**6.72E-07**
Complement component C1q receptor (CD93)	0.435	0.087	**7.32E-07**
Tumor necrosis factor receptor superfamily member 14 (TNFRSF14)	0.373	0.075	**9.81E-07**
Osteoclast-associated immunoglobulin-like receptor (hOSCAR)	0.504	0.104	**1.44E-06**
Chitinase-3-like protein 1 (CHI3L1)	0.417	0.087	**1.87E-06**
Tyrosine-protein phosphatase non-receptor type substrate 1 (SHPS-1)	0.515	0.107	**1.89E-06**
Tumor necrosis factor receptor superfamily member 10C (TNFRSF10C)	0.530	0.111	**2.11E-06**
Insulin-like growth factor-binding protein 7 (IGFBP-7)	0.412	0.087	**2.44E-06**
Fms-related tyrosine kinase 3 ligand (Flt3L)	0.468	0.100	**3.10E-06**
Interleukin-1 receptor type 1 (IL-1RT1)	0.389	0.083	**3.27E-06**
Placenta growth factor (PlGF)	0.374	0.080	**3.84E-06**
Thrombomodulin TM	0.415	0.090	**4.17E-06**
Hydroxyacid oxidase 1 (HAOX1)	0.429	0.093	**4.78E-06**
Trem-like transcript 2 protein (TLT-2)	0.436	0.095	**5.58E-06**
Cadherin-5 (CDH5)	0.393	0.087	**6.43E-06**
Resistin (RETN)	0.484	0.107	**6.53E-06**
Interleukin-1 receptor-like 2 (IL1RL2)	0.425	0.094	**6.56E-06**
Angiotensin-converting enzyme 2 (ACE2)	0.455	0.102	**9.34E-06**
CUB domain-containing protein 1 (CDCP1)	0.427	0.096	**1.03E-05**
C-C motif chemokine 4 (CCL4)	0.429	0.098	**1.42E-05**
Interleukin-12 subunit beta (IL-12B)	0.447	0.105	**2.21E-05**
Kidney injury molecule 1 (KIM-1)	0.466	0.110	**2.39E-05**
Urokinase plasminogen activator surface receptor (U-PAR)	0.375	0.089	**2.61E-05**
Peptidoglycan recognition protein 1 (PGLYRP1)	0.428	0.102	**2.77E-05**
Osteoprotegerin (OPG)	0.379	0.092	**4.32E-05**
TNF-related apoptosis-inducing ligand receptor 2 (TRAIL-R2)	0.360	0.088	**4.42E-05**

Protein	Beta	Se	P
Tumor necrosis factor receptor superfamily member 10A (TNFRSF10A)	0.346	0.084	**4.66E-05**
Granulins (GRN)	0.369	0.090	**4.96E-05**
Transferrin receptor protein 1 (TR)	0.313	0.077	**5.66E-05**
P-selectin glycoprotein ligand 1 (PSGL-1)	0.430	0.110	**9.90E-05**
Ephrin type-B receptor 4 (EPHB4)	0.318	0.082	**1.15E-04**
Low affinity immunoglobulin gamma Fc region receptor II-b (IgG Fc receptor II-b)	0.446	0.117	**1.42E-04**
Spondin-1 (SPON1)	0.292	0.077	**1.53E-04**
C-C motif chemokine 20 (CCL20)	0.299	0.081	**2.41E-04**
Fibroblast growth factor 23 (FGF-23)	0.286	0.080	**3.42E-04**
Oncostatin-M (OSM)	0.285	0.080	**4.01E-04**
Signaling lymphocytic activation molecule (SLAMF1)	0.337	0.095	**4.28E-04**
Epithelial cell adhesion molecule (Ep-CAM)	-0.323	0.092	**4.54E-04**
Urokinase-type plasminogen activator (uPA)	0.304	0.088	**5.28E-04**
Tumor necrosis factor (Ligand) superfamily, member 12 (TWEAK)	-0.289	0.084	**6.35E-04**
Tissue factor pathway inhibitor (TFPI)	0.309	0.091	**6.64E-04**
Thrombospondin-2 (THBS2)	0.299	0.089	**8.40E-04**
Vascular endothelial growth factor D (VEGF-D)	-0.353	0.106	**9.23E-04**
C-C motif chemokine 19 (CCL19)	0.321	0.097	**9.30E-04**
C-X-C motif chemokine 10 (CXCL10)	0.295	0.089	**1.01E-03**
Growth/differentiation factor 15 (GDF-15)	0.280	0.089	**1.70E-03**
Monocyte chemotactic protein 3 (MCP-3)	0.343	0.109	**1.71E-03**
Tyrosine-protein kinase Mer (MERTK)	0.259	0.084	**2.20E-03**
Tumor necrosis factor receptor superfamily member 13B (TNFRSF13B)	0.279	0.091	**2.24E-03**
Neurotrophin-3 (NT-3)	-0.230	0.076	**2.56E-03**
Poly [ADP-ribose] polymerase 1 (PARP-1)	0.227	0.078	**3.47E-03**
Beta-nerve growth factor (Beta-NGF)	-0.254	0.088	**3.94E-03**
TNF-related activation-induced cytokine (TRANCE)	0.261	0.091	**4.09E-03**
Programmed cell death 1 ligand 1 (PD-L1)	0.251	0.089	**5.09E-03**
Prolargin (PRELP)	0.240	0.088	**6.44E-03**
C-C motif chemokine 15 (CCL15)	0.266	0.098	**6.58E-03**

Protein	Beta	Se	P
T-cell surface glycoprotein CD5 (CD5)	0.259	0.095	**6.68E-03**
Interleukin-6 receptor subunit alpha (IL-6RA)	0.288	0.109	**8.34E-03**
Matrix metalloproteinase-9 (MMP-9)	0.205	0.079	**9.71E-03**
Lectin-like oxidized LDL receptor 1 (LOX-1)	0.203	0.080	**1.17E-02**
Carcinoembryonic antigenrelated cell adhesion molecule 8 (CEACAM8)	0.234	0.094	**1.30E-02**
Matrix metalloproteinase-3 (MMP-3)	0.282	0.115	**1.47E-02**
Cystatin-B (CSTB)	0.194	0.080	**1.56E-02**
Chymotrypsin C (CTRC)	-0.228	0.097	**1.87E-02**
C-C motif chemokine 17 (CCL17)	0.191	0.082	**1.96E-02**
P-selectin (SELP)	0.171	0.073	**1.96E-02**
Proheparin-binding EGF-like growth factor (HB-EGF)	0.170	0.073	**2.01E-02**
Intercellular adhesion molecule 2 (ICAM-2)	0.229	0.099	**2.07E-02**
Lymphotactin (XCL1)	0.231	0.101	**2.32E-02**
Artemin (ARTN)	-0.271	0.121	2.54E-02
Leukemia inhibitory factor (LIF)	0.391	0.175	2.55E-02
Interleukin-15 receptor subunit alpha (IL-15RA)	0.174	0.080	2.91E-02
Interleukin-10 (IL-10)	0.179	0.083	3.10E-02
Thrombopoietin (THPO)	0.162	0.076	3.37E-02
Interleukin-17C (IL-17C)	0.195	0.092	3.44E-02
Interleukin-7 (IL-7)	0.162	0.077	3.48E-02
C-C motif chemokine 22 (CCL22)	0.237	0.113	3.55E-02
C-C motif chemokine 23 (CCL23)	-0.194	0.092	3.56E-02
Interleukin-5 (IL-5)	0.317	0.152	3.80E-02
Galectin-4 (Gal-4)	0.179	0.087	4.04E-02
T-cell surface glycoprotein CD4 (CD4)	0.172	0.084	4.06E-02
von Willebrand factor (vWF)	0.142	0.070	4.13E-02
Cathepsin L1 (CTSL1)	0.181	0.089	4.28E-02
Protein S100-A12 (EN-RAGE)	0.165	0.081	4.31E-02
Matrix extracellular phosphoglycoprotein (MEPE)	0.190	0.094	4.37E-02
Epidermal growth factor receptor (EGFR)	0.151	0.076	4.67E-02

**Protein**	**Beta**	**Se**	**P**
Follistatin (FS)	0.147	0.075	5.03E-02
Leukemia inhibitory factor receptor (LIF-R)	-0.155	0.082	5.88E-02
Brother of CDO (Protein BOC)	0.154	0.083	6.31E-02
Fibroblast growth factor 19 (FGF-19)	-0.133	0.072	6.61E-02
Natriuretic peptides B (BNP)	0.172	0.096	7.39E-02
Bleomycin hydrolase (BLM hydrolase)	0.136	0.076	7.47E-02
Glial cell line-derived neurotrophic factor (GDNF)	0.148	0.084	7.97E-02
Interleukin-20 (IL-20)	0.311	0.178	8.13E-02
Latency-associated peptide transforming growth factor beta-1 (LAP TGF-beta-1)	0.129	0.074	8.35E-02
Carboxypeptidase B (CPB1)	0.170	0.099	8.46E-02
Transforming growth factor alpha (TGF-alpha)	0.132	0.077	8.61E-02
Chitotriosidase-1 (CHIT1)	0.227	0.134	9.16E-02
Decorin (DCN)	-0.145	0.088	9.80E-02
TNF-related apoptosis-inducing ligand (TRAIL)	0.137	0.084	1.01E-01
Galectin-3 (Gal-3)	0.135	0.083	1.04E-01
Interleukin-27 (IL-27)	-0.164	0.102	1.10E-01
Matrix metalloproteinase-7 (MMP-7)	0.144	0.092	1.16E-01
Proteinase-activated receptor 1 (PAR-1)	0.109	0.069	1.16E-01
TNF-beta (TNFB)	0.160	0.103	1.21E-01
Dickkopf-related protein 1 (Dkk-1)	0.119	0.077	1.21E-01
Matrix metalloproteinase-2 (MMP-2)	0.122	0.080	1.26E-01
Stem cell factor (SCF)	-0.147	0.097	1.27E-01
ST2 protein (ST2)	0.169	0.111	1.29E-01
Collagen alpha-1(I) chain (COL1A1)	-0.134	0.089	1.34E-01
C-C motif chemokine 28 (CCL28)	-0.133	0.093	1.52E-01
Platelet-derived growth factor subunit A (PDGF subunit A)	0.104	0.073	1.58E-01
Receptor for advanced glycosylation end products (RAGE)	-0.140	0.099	1.59E-01
C-X-C motif chemokine 11 (CXCL11)	0.123	0.090	1.71E-01
Superoxide dismutase [Mn], mitochondrial (SOD2)	0.101	0.074	1.74E-01
Pulmonary surfactant-associated protein D (PSP-D)	0.139	0.105	1.85E-01

Protein	Beta	Se	P
A disintegrin and metalloproteinase with thrombospondin motifs 13 (ADAM-TS13)	0.113	0.086	1.88E-01
Melusin (ITGB1BP2)	0.089	0.069	1.93E-01
Platelet endothelial cell adhesion molecule (PECAM-1)	0.092	0.071	1.99E-01
Interleukin-4 receptor subunit alpha (IL-4RA)	0.100	0.079	2.05E-01
Sortilin (SORT1)	0.100	0.080	2.08E-01
Platelet-derived growth factor subunit B (PDGF subunit B)	0.092	0.074	2.12E-01
Programmed cell death 1 ligand 2 (PD-L2)	-0.118	0.095	2.12E-01
T cell surface glycoprotein CD6 isoform (CD6)	0.125	0.104	2.31E-01
CD40 ligand (CD40-L)	0.080	0.067	2.35E-01
Interleukin-20 receptor subunit alpha (IL-20RA)	-0.219	0.186	2.40E-01
Osteopontin (OPN)	-0.102	0.088	2.49E-01
C-X-C motif chemokine 6 (CXCL6)	0.094	0.082	2.56E-01
Neurturin (NRTN)	-0.190	0.168	2.58E-01
C-X-C motif chemokine 5 (CXCL5)	0.091	0.083	2.70E-01
SLAM family member 7 (SLAMF7)	0.110	0.100	2.71E-01
Interleukin-24 (IL-24)	0.179	0.166	2.81E-01
Monocyte chemotactic protein 2 (MCP-2)	0.104	0.098	2.87E-01
Trefoil factor 3 (TFF3)	0.097	0.092	2.90E-01
Thymic stromal lymphopoietin (TSLP)	0.438	0.419	2.97E-01
Fatty acid-binding protein, intestinal (FABP2)	0.081	0.079	3.09E-01
Delta and Notch-like epidermal growth factor-related receptor (DNER)	-0.103	0.102	3.10E-01
Eotaxin-1 (CCL11)	-0.094	0.095	3.20E-01
Matrix metalloproteinase-10 (MMP-10)	-0.083	0.085	3.28E-01
Carboxypeptidase A1 (CPA1)	0.095	0.098	3.29E-01
Sulfotransferase 1A1 (ST1A1)	0.072	0.075	3.38E-01
Heme oxygenase 1 (HO-1)	0.085	0.096	3.75E-01
Caspase-8 (CASP-8)	0.064	0.073	3.81E-01
SLAM family member 5 (CD84)	0.063	0.073	3.92E-01
Serine/threonine-protein kinase 4 (STK4)	0.059	0.069	3.92E-01
Brain-derived neurotrophic factor (BDNF)	0.113	0.133	3.93E-01

Protein	Beta	Se	P
Kallikrein-6 (KLK6)	0.064	0.077	4.10E-01
Serine protease 27 (PRSS27)	0.084	0.103	4.14E-01
Interleukin-17 receptor A (IL-17RA)	0.079	0.103	4.40E-01
Interleukin-4 (IL-4)	0.111	0.145	4.42E-01
Neurogenic locus notch homolog protein 3 (Notch 3)	0.070	0.093	4.49E-01
Angiopoietin-1 (ANG-1)	0.056	0.074	4.50E-01
Tissue factor (TF)	-0.072	0.098	4.61E-01
Growth/differentiation factor 2 (GDF-2)	-0.067	0.091	4.63E-01
Heat shock 27 kDa protein (HSP 27)	0.048	0.067	4.76E-01
Interferon gamma (IFN-gamma)	-0.288	0.405	4.77E-01
Gastric intrinsic factor (GIF)	0.077	0.110	4.86E-01
Junctional adhesion molecule A (JAM-A)	0.048	0.072	5.04E-01
Bone morphogenetic protein 6 (BMP-6)	0.053	0.082	5.17E-01
Matrix metalloproteinase-12 (MMP-12)	0.059	0.096	5.36E-01
C-X-C motif chemokine 1 (CXCL1)	0.047	0.078	5.48E-01
Caspase-3 (CASP-3)	0.040	0.067	5.57E-01
Polymeric immunoglobulin receptor (PIgR)	0.053	0.091	5.61E-01
STAM-binding protein (STAMPB)	-0.038	0.067	5.66E-01
Lactoylglutathione lyase (GLO1)	0.038	0.066	5.67E-01
Aminopeptidase N (AP-N)	-0.051	0.092	5.80E-01
Interleukin-17D (IL-17D)	0.043	0.080	5.88E-01
Interleukin-1 alpha (IL-1 alpha)	0.121	0.239	6.12E-01
Renin (REN)	0.045	0.088	6.14E-01
Angiopoietin-1 receptor (TIE2)	0.046	0.092	6.17E-01
2,4-dienoyl-CoA reductase, mitochondrial (DECR1)	0.034	0.069	6.18E-01
Proto-oncogene tyrosine-protein kinase Src (SRC)	0.031	0.064	6.26E-01
Interleukin-8 (IL-8)	0.036	0.081	6.52E-01
V-set and immunoglobulin domain-containing protein 2 (VSIG2)	-0.036	0.084	6.65E-01
Adenosine Deaminase (ADA)	0.032	0.079	6.81E-01
Serpin A12 (SERPINA12)	0.037	0.103	7.16E-01

Protein	Beta	Se	P
Tumor necrosis factor (TNF)	0.062	0.185	7.39E-01
Interleukin-33 (IL-33)	-0.089	0.283	7.52E-01
Protein-glutamine gamma-glutamyltransferase 2 (TGM2)	-0.020	0.064	7.55E-01
Fractalkine (CX3CL1)	-0.028	0.093	7.60E-01
NF-kappa-B essential modulator (NEMO)	0.020	0.069	7.74E-01
Tumor necrosis factor receptor superfamily member 9 (TNFRSF9)	0.026	0.096	7.90E-01
Cystatin D (CST5)	-0.026	0.097	7.91E-01
Gastrotropin (GT)	-0.021	0.085	8.05E-01
Agouti-related protein (AGRP)	0.019	0.079	8.08E-01
Contactin-1 (CNTN1)	-0.020	0.087	8.21E-01
Interleukin-2 receptor subunit beta (IL-2RB)	-0.045	0.203	8.23E-01
Interleukin-10 receptor subunit alpha (IL-10RA)	-0.026	0.141	8.56E-01
CD40L receptor (CD40)	-0.012	0.073	8.69E-01
Axin-1 (AXIN1)	-0.011	0.069	8.73E-01
Interleukin-17A (IL-17A)	0.012	0.102	9.06E-01
C-C motif chemokine 25 (CCL25)	-0.009	0.103	9.28E-01
Interleukin-13 (IL-13)	0.013	0.171	9.38E-01
C-C motif chemokine 24 (CCL24)	0.009	0.120	9.44E-01
SIR2-like protein 2 (SIRT2)	0.004	0.067	9.49E-01
Natural killer cell receptor 2B4 (CD244)	-0.005	0.079	9.52E-01
Fibroblast growth factor 5 (FGF-5)	0.006	0.099	9.53E-01
C-X-C motif chemokine 9 (CXCL9)	-0.005	0.089	9.56E-01
Matrix metalloproteinase-1 (MMP-1)	-0.005	0.091	9.57E-01
Pappalysin-1 (PAPPA)	-0.004	0.094	9.63E-01
Monocyte chemotactic protein 4 (MCP-4)	0.002	0.089	9.84E-01
Eukaryotic translation initiation factor 4E-binding protein 1 (4E-BP1)	-0.001	0.065	9.88E-01

Supplementary Table 3. Associations between blood protein levels at baseline and weight loss (Δ BMI= $BMI_{3months}$ - $BMI_{baseline}$).

Data are from linear regression analyses adjusted for age, sex and race.

Protein	Beta	Se	P
Fibroblast growth factor 21 (FGF-21)	-0.281	0.062	**7.22E-06**
Spondin-2 (SPON2)	-0.198	0.063	1.62E-03
Plasminogen activator inhibitor 1 (PAI)	-0.198	0.063	1.66E-03
Matrix extracellular phosphoglycoprotein (MEPE)	-0.215	0.069	1.79E-03
Brother of CDO (Protein BOC)	-0.191	0.063	2.57E-03
Poly [ADP-ribose] polymerase 1 (PARP-1)	-0.194	0.064	2.65E-03
Fibroblast growth factor 23 (FGF-23)	-0.172	0.062	6.11E-03
E-selectin (SELE)	-0.177	0.065	6.15E-03
Fatty acid-binding protein, adipocyte (FABP4)	-0.178	0.065	6.30E-03
Monocyte chemotactic protein 3 (MCP-3)	-0.199	0.077	9.93E-03
C-X-C motif chemokine 9 (CXCL9)	0.164	0.064	1.01E-02
Urokinase plasminogen activator surface receptor (U-PAR)	-0.165	0.065	1.17E-02
C-X-C motif chemokine 11 (CXCL11)	0.159	0.063	1.22E-02
Interleukin-1 receptor antagonist protein (IL-1ra)	-0.156	0.062	1.30E-02
Trefoil factor 3 (TFF3)	0.150	0.064	1.87E-02
Bone morphogenetic protein 6 (BMP-6)	-0.145	0.062	1.96E-02
Heme oxygenase 1 (HO-1)	-0.149	0.065	2.30E-02
TNF-related activation-induced cytokine (TRANCE)	-0.144	0.064	2.34E-02
Tissue-type plasminogen activator (t-PA)	-0.149	0.066	2.49E-02
Tumor necrosis factor receptor superfamily member 11A (TNFRSF11A)	-0.140	0.063	2.57E-02
Agouti-related protein (AGRP)	-0.149	0.067	2.68E-02
Serine protease 27 (PRSS27)	-0.139	0.063	2.72E-02
Interleukin-1 receptor-like 2 (IL1RL2)	-0.135	0.062	2.92E-02
Interferon gamma (IFN-gamma)	0.762	0.349	2.97E-02
Lactoylglutathione lyase (GLO1)	-0.132	0.062	3.22E-02
Tyrosine-protein phosphatase non-receptor type substrate 1 (SHPS-1)	-0.133	0.064	3.76E-02
Tumor necrosis factor receptor 1 (TNF-R1)	-0.131	0.065	4.40E-02

Protein	Beta	Se	P
T-cell surface glycoprotein CD4 (CD4)	-0.126	0.063	4.58E-02
Matrix metalloproteinase-2 (MMP-2)	-0.122	0.062	5.01E-02
Prolargin (PRELP)	-0.125	0.064	5.11E-02
ADM (ADM)	-0.125	0.065	5.46E-02
Thrombomodulin TM	-0.125	0.065	5.46E-02
Insulin-like growth factor-binding protein 7 (IGFBP-7)	-0.132	0.069	5.61E-02
Ephrin type-B receptor 4 (EPHB4)	-0.123	0.065	5.70E-02
Decorin (DCN)	-0.123	0.065	5.79E-02
CUB domain-containing protein 1 (CDCP1)	-0.120	0.063	5.85E-02
Eukaryotic translation initiation factor 4E-binding protein 1 (4E-BP1)	-0.119	0.063	5.93E-02
Insulin-like growth factor-binding protein 1 (IGFBP-1)	0.122	0.065	6.29E-02
Collagen alpha-1(I) chain (COL1A1)	-0.120	0.065	6.66E-02
Interleukin-4 receptor subunit alpha (IL-4RA)	-0.114	0.063	6.81E-02
Cathepsin Z (CTSZ)	-0.116	0.064	6.88E-02
Tartrate-resistant acid phosphatase type 5 (TR-AP)	-0.123	0.067	6.94E-02
Cathepsin D (CTSD)	-0.125	0.070	7.55E-02
Azurocidin (AZU1	-0.111	0.063	7.82E-02
Interleukin-17D (IL-17D)	-0.112	0.064	8.06E-02
Junctional adhesion molecule A (JAM-A)	0.108	0.062	8.08E-02
Axin-1 (AXIN1)	0.109	0.063	8.25E-02
Angiopoietin-1 receptor (TIE2)	-0.109	0.064	9.03E-02
Galectin-9 (Gal-9)	-0.104	0.062	9.36E-02
Oncostatin-M (OSM)	-0.105	0.063	9.71E-02
Hepatocyte growth factor (HGF)	-0.107	0.065	1.03E-01
C-X-C motif chemokine 16 (CXCL16)	-0.104	0.064	1.07E-01
Thrombospondin-2 (THBS2)	-0.101	0.062	1.07E-01
Leukemia inhibitory factor receptor (LIF-R)	-0.100	0.064	1.16E-01
Placenta growth factor (PlGF)	-0.104	0.066	1.17E-01
Pentraxin-related protein PTX3 (PTX3)	-0.097	0.063	1.21E-01
Vascular endothelial growth factor A (VEGF-A)	0.097	0.063	1.23E-01

Protein	Beta	Se	P
Programmed cell death 1 ligand 2 (PD-L2)	-0.094	0.062	1.29E-01
Cadherin-5 (CDH5)	-0.094	0.062	1.32E-01
Tumor necrosis factor receptor superfamily member 9 (TNFRSF9)	0.101	0.067	1.33E-01
Integrin beta-2 (ITGB2)	-0.092	0.062	1.41E-01
Protein S100-A12 (EN-RAGE)	0.093	0.063	1.42E-01
Complement component C1q receptor (CD93)	-0.092	0.064	1.53E-01
Lipoprotein lipase (LPL)	-0.099	0.070	1.58E-01
Myeloblastin (PRTN3)	-0.088	0.063	1.59E-01
Monocyte chemotactic protein 1 (MCP-1)	-0.086	0.062	1.69E-01
Carbonic anhydrase 5A, mitochondrial (CA5A)	-0.091	0.066	1.70E-01
SLAM family member 5 (CD84)	-0.085	0.062	1.72E-01
Cystatin D (CST5)	0.088	0.065	1.76E-01
Protein-glutamine gamma-glutamyltransferase 2 (TGM2)	-0.084	0.062	1.78E-01
Perlecan (PLC)	-0.087	0.065	1.81E-01
Interleukin-2 receptor subunit alpha (IL2-RA)	0.089	0.066	1.82E-01
Proto-oncogene tyrosine-protein kinase Src (SRC)	0.081	0.062	1.91E-01
Neurturin (NRTN)	0.139	0.106	1.92E-01
Osteoprotegerin (OPG)	0.084	0.065	1.97E-01
Interleukin-20 (IL-20)	-0.137	0.106	1.98E-01
Caspase-3 (CASP-3)	0.079	0.062	1.99E-01
Tyrosine-protein kinase receptor UFO (AXL)	-0.085	0.067	2.04E-01
TNF-beta (TNFB)	-0.080	0.063	2.07E-01
C-X-C motif chemokine 10 (CXCL10)	0.078	0.062	2.08E-01
Interleukin-4 (IL-4)	-0.111	0.088	2.09E-01
Tumor necrosis factor receptor superfamily member 10A (TNFRSF10A)	0.077	0.062	2.16E-01
Osteoclast-associated immunoglobulin-like receptor (hOSCAR)	-0.079	0.065	2.19E-01
Caspase-8 (CASP-8)	-0.078	0.063	2.20E-01
Interleukin-2 receptor subunit beta (IL-2RB)	0.160	0.131	2.24E-01
Leptin (LEP)	-0.102	0.084	2.26E-01
Eotaxin-1 (CCL11)	0.079	0.066	2.30E-01

Protein	Beta	Se	P
Serpin A12 (SERPINA12)	-0.078	0.065	2.31E-01
Pappalysin-1 (PAPPA)	-0.083	0.070	2.32E-01
Proprotein convertase subtilisin/kexin type 9 (PCSK9)	0.075	0.063	2.34E-01
Myoglobin (MB)	-0.089	0.074	2.35E-01
Matrix metalloproteinase-10 (MMP-10)	0.076	0.064	2.39E-01
Vascular endothelial growth factor D (VEGF-D)	-0.075	0.064	2.44E-01
Macrophage colony-stimulating factor 1 (CSF-1)	0.076	0.065	2.44E-01
Natriuretic peptides B (BNP)	-0.114	0.098	2.46E-01
Prostasin (PRSS8)	-0.075	0.065	2.47E-01
CD40L receptor (CD40)	0.072	0.062	2.47E-01
Contactin-1 (CNTN1)	-0.071	0.062	2.51E-01
Lectin-like oxidized LDL receptor 1 (LOX-1)	-0.070	0.062	2.59E-01
Cystatin-B (CSTB)	-0.070	0.063	2.60E-01
Interleukin-1 receptor type 2 (IL-1RT2)	-0.072	0.065	2.64E-01
Angiopoietin-1 (ANG-1)	-0.068	0.061	2.66E-01
Tumor necrosis factor receptor superfamily member 6 (FAS)	-0.076	0.068	2.67E-01
Leukemia inhibitory factor (LIF)	0.128	0.116	2.67E-01
Resistin (RETN)	0.071	0.064	2.68E-01
Alpha-L-iduronidase (IDUA)	-0.069	0.062	2.68E-01
Programmed cell death 1 ligand 1 (PD-L1)	0.072	0.065	2.71E-01
Proheparin-binding EGF-like growth factor (HB-EGF)	-0.067	0.061	2.73E-01
Carcinoembryonic antigenrelated cell adhesion molecule 8 (CEACAM8)	-0.069	0.063	2.75E-01
Elafin (PI3)	-0.071	0.065	2.75E-01
Polymeric immunoglobulin receptor (PIgR)	-0.072	0.066	2.77E-01
Pro-interleukin-16 (IL16)	-0.069	0.064	2.85E-01
Interleukin-5 (IL-5)	-0.093	0.087	2.86E-01
A disintegrin and metalloproteinase with thrombospondin motifs 13 (ADAM-TS13)	-0.065	0.061	2.87E-01
NF-kappa-B essential modulator (NEMO)	0.066	0.063	2.95E-01
Sulfotransferase 1A1 (ST1A1)	0.065	0.062	2.98E-01
C-C motif chemokine 24 (CCL24)	0.067	0.064	3.01E-01

Protein	Beta	Se	P
Low-density lipoprotein receptor (LDL receptor)	-0.064	0.062	3.09E-01
Interleukin-17 receptor A (IL-17RA)	-0.063	0.062	3.13E-01
Matrix metalloproteinase-9 (MMP-9)	-0.063	0.062	3.14E-01
Serine/threonine-protein kinase 4 (STK4)	0.060	0.061	3.25E-01
Aminopeptidase N (AP-N)	-0.064	0.065	3.28E-01
Interleukin-8 (IL-8)	0.061	0.063	3.29E-01
TNF-related apoptosis-inducing ligand (TRAIL)	-0.063	0.065	3.31E-01
Interleukin-12 subunit beta (IL-12B)	0.062	0.064	3.34E-01
Interleukin-18-binding protein (IL-18BP)	-0.064	0.067	3.34E-01
Spondin-1 (SPON1)	-0.061	0.063	3.37E-01
Interleukin-6 receptor subunit alpha (IL-6RA)	-0.061	0.064	3.40E-01
Tissue factor pathway inhibitor (TFPI)	-0.063	0.067	3.46E-01
Interleukin-6 (IL-6)	-0.058	0.062	3.48E-01
ST2 protein (ST2)	-0.065	0.069	3.51E-01
Transferrin receptor protein 1 (TR)	-0.057	0.061	3.54E-01
Neurogenic locus notch homolog protein 3 (Notch 3)	-0.059	0.064	3.54E-01
Monocyte chemotactic protein 4 (MCP-4)	0.058	0.063	3.57E-01
von Willebrand factor (vWF)	0.057	0.062	3.61E-01
Interleukin-27 (IL-27)	0.058	0.064	3.63E-01
C-C motif chemokine 17 (CCL17)	0.057	0.063	3.65E-01
Peptidoglycan recognition protein 1 (PGLYRP1)	-0.058	0.065	3.79E-01
Tumor necrosis factor receptor 2 (TNF-R2)	-0.056	0.065	3.87E-01
Growth hormone (GH)	-0.060	0.070	3.91E-01
Tumor necrosis factor (Ligand) superfamily, member 12 (TWEAK)	0.054	0.063	3.91E-01
C-C motif chemokine 19 (CCL19)	-0.053	0.062	3.94E-01
Fibroblast growth factor 19 (FGF-19)	0.053	0.062	3.96E-01
Matrix metalloproteinase-7 (MMP-7)	-0.053	0.063	4.06E-01
Tissue factor (TF)	-0.054	0.065	4.09E-01
Kallikrein-6 (KLK6)	-0.051	0.062	4.11E-01
Interleukin-18 receptor 1 (IL-18R1)	-0.051	0.063	4.19E-01

Protein	Beta	Se	P
Artemin (ARTN)	0.074	0.095	4.39E-01
Platelet endothelial cell adhesion molecule (PECAM-1)	0.048	0.062	4.40E-01
Tyrosine-protein kinase Mer (MERTK)	-0.049	0.064	4.43E-01
Interleukin-17C (IL-17C)	-0.060	0.078	4.47E-01
Superoxide dismutase [Mn], mitochondrial (SOD2)	-0.048	0.064	4.55E-01
Natural killer cell receptor 2B4 (CD244)	0.046	0.062	4.56E-01
C-C motif chemokine 15 (CCL15)	-0.047	0.063	4.59E-01
C-C motif chemokine 3 (CCL3)	-0.047	0.064	4.62E-01
P-selectin glycoprotein ligand 1 (PSGL-1)	-0.047	0.064	4.63E-01
Tumor necrosis factor ligand superfamily member 13B (TNFSF13B)	-0.046	0.063	4.67E-01
Lymphotoxin-beta receptor (LTBR)	-0.047	0.066	4.71E-01
Interleukin-18 (IL-18)	-0.046	0.065	4.76E-01
Growth/differentiation factor 15 (GDF-15)	0.047	0.066	4.79E-01
Interleukin-1 receptor type 1 (IL-1RT1)	0.043	0.062	4.92E-01
Bleomycin hydrolase (BLM hydrolase)	-0.044	0.064	4.95E-01
Interleukin-13 (IL-13)	-0.071	0.104	4.96E-01
Cathepsin L1 (CTSL1)	0.043	0.064	4.99E-01
Delta and Notch-like epidermal growth factor-related receptor (DNER)	-0.047	0.070	5.02E-01
C-C motif chemokine 28 (CCL28)	-0.043	0.066	5.14E-01
Neurotrophin-3 (NT-3)	0.041	0.063	5.16E-01
C-C motif chemokine 16 (CCL16)	-0.042	0.066	5.21E-01
Protein delta homolog 1 (DLK-1)	-0.042	0.066	5.28E-01
Carboxypeptidase A1 (CPA1)	0.038	0.063	5.47E-01
Tumor necrosis factor receptor superfamily member 13B (TNFRSF13B)	0.038	0.064	5.48E-01
Tumor necrosis factor receptor superfamily member 10C (TNFRSF10C)	-0.038	0.063	5.53E-01
Urokinase-type plasminogen activator (uPA)	-0.037	0.063	5.62E-01
Thymic stromal lymphopoietin (TSLP)	0.188	0.336	5.76E-01
Interleukin-17A (IL-17A)	0.045	0.080	5.78E-01
C-C motif chemokine 20 (CCL20)	-0.035	0.063	5.78E-01
Epidermal growth factor receptor (EGFR)	0.035	0.063	5.81E-01

Protein	Beta	Se	P
Dickkopf-related protein 1 (Dkk-1)	-0.033	0.062	5.93E-01
Metalloproteinase inhibitor 4 (TIMP4)	-0.035	0.067	5.97E-01
C-C motif chemokine 22 (CCL22)	-0.033	0.063	6.02E-01
Chitotriosidase-1 (CHIT1)	0.035	0.067	6.02E-01
Insulin-like growth factor-binding protein 2 (IGFBP-2)	0.033	0.064	6.03E-01
V-set and immunoglobulin domain-containing protein 2 (VSIG2)	-0.032	0.063	6.10E-01
Follistatin (FS)	0.032	0.062	6.10E-01
Heat shock 27 kDa protein (HSP 27)	0.033	0.064	6.11E-01
Hydroxyacid oxidase 1 (HAOX1)	-0.034	0.068	6.11E-01
Carboxypeptidase B (CPB1)	0.032	0.064	6.23E-01
Paraoxonase (PON3)	0.029	0.062	6.35E-01
Growth/differentiation factor 2 (GDF-2)	0.031	0.066	6.36E-01
C-X-C motif chemokine 5 (CXCL5)	-0.029	0.063	6.50E-01
Interleukin-20 receptor subunit alpha (IL-20RA)	-0.048	0.108	6.57E-01
Tumor necrosis factor (TNF)	-0.045	0.104	6.62E-01
Galectin-4 (Gal-4)	0.027	0.061	6.62E-01
Osteopontin (OPN)	0.028	0.063	6.64E-01
Epithelial cell adhesion molecule (Ep-CAM)	0.027	0.062	6.67E-01
C-C motif chemokine 25 (CCL25)	0.027	0.064	6.76E-01
Gastrotropin (GT)	0.027	0.065	6.78E-01
Protein AMBP (AMBP)	-0.027	0.065	6.79E-01
2,4-dienoyl-CoA reductase, mitochondrial (DECR1)	-0.025	0.062	6.85E-01
Matrix metalloproteinase-12 (MMP-12)	-0.024	0.061	6.92E-01
Chitinase-3-like protein 1 (CHI3L1)	0.025	0.062	6.93E-01
Stem cell factor (SCF)	-0.025	0.064	6.95E-01
Receptor for advanced glycosylation end products (RAGE)	-0.025	0.064	6.97E-01
Transforming growth factor alpha (TGF-alpha)	-0.025	0.064	6.99E-01
Pulmonary surfactant-associated protein D (PSP-D)	-0.026	0.067	7.00E-01
Chymotrypsin C (CTRC)	0.025	0.065	7.01E-01
Monocyte chemotactic protein 2 (MCP-2)	0.023	0.064	7.18E-01

Protein	Beta	Se	P
CD40 ligand (CD40-L)	-0.022	0.062	7.19E-01
Intercellular adhesion molecule 2 (ICAM-2)	-0.022	0.062	7.20E-01
Thrombopoietin (THPO)	0.022	0.062	7.21E-01
C-C motif chemokine 23 (CCL23)	0.023	0.066	7.29E-01
Renin (REN)	-0.021	0.063	7.38E-01
Gastric intrinsic factor (GIF)	0.021	0.063	7.38E-01
Interleukin-10 receptor subunit alpha (IL-10RA)	0.024	0.084	7.75E-01
Interleukin-24 (IL-24)	0.029	0.107	7.85E-01
Matrix metalloproteinase-1 (MMP-1)	-0.017	0.063	7.88E-01
SLAM family member 7 (SLAMF7)	0.020	0.076	7.91E-01
Adenosine Deaminase (ADA)	-0.017	0.063	7.91E-01
CD166 antigen (ALCAM)	-0.017	0.065	7.97E-01
Signaling lymphocytic activation molecule (SLAMF1)	-0.016	0.064	8.00E-01
TNF-related apoptosis-inducing ligand receptor 2 (TRAIL-R2)	-0.016	0.064	8.03E-01
Granulins (GRN)	-0.015	0.061	8.10E-01
Brain-derived neurotrophic factor (BDNF)	-0.016	0.067	8.11E-01
Fibroblast growth factor 5 (FGF-5)	-0.017	0.071	8.12E-01
Myeloperoxidase (MPO)	0.014	0.062	8.19E-01
Platelet-derived growth factor subunit A (PDGF subunit A)	-0.014	0.062	8.21E-01
Matrix metalloproteinase-3 (MMP-3)	0.018	0.079	8.23E-01
P-selectin (SELP)	0.014	0.062	8.24E-01
Sortilin (SORT1)	-0.014	0.063	8.27E-01
Galectin-3 (Gal-3)	0.014	0.064	8.32E-01
Secretoglobin family 3A member 2 (SCGB3A2)	-0.013	0.062	8.32E-01
Macrophage receptor MARCO (MARCO)	-0.012	0.062	8.46E-01
Kidney injury molecule 1 (KIM-1)	-0.012	0.067	8.53E-01
T-cell surface glycoprotein CD5 (CD5)	0.011	0.064	8.59E-01
Low affinity immunoglobulin gamma Fc region receptor II-b (IgG Fc receptor II-b)	-0.011	0.063	8.64E-01
Glial cell line-derived neurotrophic factor (GDNF)	0.012	0.068	8.64E-01
Fatty acid-binding protein, intestinal (FABP2)	-0.010	0.062	8.70E-01

Protein	Beta	Se	P
STAM-binding protein (STAMPB)	0.010	0.063	8.74E-01
Beta-nerve growth factor (Beta-NGF)	0.010	0.063	8.75E-01
C-X-C motif chemokine 6 (CXCL6)	-0.009	0.064	8.82E-01
SIR2-like protein 2 (SIRT2)	0.009	0.063	8.85E-01
Angiotensin-converting enzyme 2 (ACE2)	-0.010	0.070	8.87E-01
Latency-associated peptide transforming growth factor beta-1 (LAP TGF-beta-1)	-0.009	0.062	8.88E-01
C-C motif chemokine 4 (CCL4)	-0.009	0.065	8.90E-01
Proteinase-activated receptor 1 (PAR-1)	0.008	0.063	8.97E-01
Platelet-derived growth factor subunit B (PDGF subunit B)	-0.007	0.061	9.02E-01
Lymphotactin (XCL1)	-0.008	0.062	9.03E-01
Interleukin-10 (IL-10)	0.007	0.064	9.11E-01
Interleukin-10 receptor subunit beta (IL-10RB)	0.006	0.066	9.24E-01
Interleukin-15 receptor subunit alpha (IL-15RA)	-0.006	0.065	9.30E-01
Retinoic acid receptor responder protein 2 (RARRES2)	-0.005	0.063	9.39E-01
T cell surface glycoprotein CD6 isoform (CD6)	0.005	0.063	9.41E-01
Fractalkine (CX3CL1)	-0.004	0.064	9.52E-01
Trem-like transcript 2 protein (TLT-2)	-0.004	0.067	9.56E-01
Interleukin-7 (IL-7)	0.003	0.061	9.62E-01
Scavenger receptor cysteine-rich type 1 protein M130 (CD163)	-0.002	0.062	9.69E-01
Tumor necrosis factor ligand superfamily member 14 (TNFSF14)	-0.002	0.063	9.76E-01
C-X-C motif chemokine 1 (CXCL1)	-0.002	0.062	9.78E-01
Fms-related tyrosine kinase 3 ligand (Flt3L)	-0.001	0.064	9.85E-01
Melusin (ITGB1BP2)	0.001	0.062	9.88E-01
Interleukin-1 alpha (IL-1 alpha)	0.003	0.219	9.90E-01
Tumor necrosis factor receptor superfamily member 14 (TNFRSF14)	0.001	0.062	9.90E-01
Interleukin-33 (IL-33)	0.001	0.157	9.97E-01

Supplementary Table 4. Interaction effect of blood protein levels at baseline and diet on weight loss (Δ BMI= $BMI_{3months}$ - $BMI_{baseline}$).

Data are from linear regression analyses including protein*diet interaction term, adjusted for age, sex and race.

**Name**	**Beta**	**Se**	**P**
Matrix metalloproteinase-7 (MMP-7)	0.371	0.123	2.61E-03
Metalloproteinase inhibitor 4 (TIMP4)	0.323	0.125	1.00E-02
Galectin-9 (Gal-9)	0.302	0.120	1.21E-02
Neurturin (NRTN)	-0.522	0.210	1.34E-02
Renin (REN)	0.281	0.124	2.38E-02
Cathepsin D (CTSD)	0.291	0.135	3.15E-02
Interleukin-24 (IL-24)	-0.441	0.212	3.76E-02
Adenosine Deaminase (ADA)	-0.250	0.122	4.09E-02
Insulin-like growth factor-binding protein 2 (IGFBP-2)	0.250	0.124	4.32E-02
Fatty acid-binding protein, intestinal (FABP2)	0.243	0.122	4.72E-02
Low-density lipoprotein receptor (LDL receptor)	0.242	0.122	4.74E-02
Proto-oncogene tyrosine-protein kinase Src (SRC)	-0.243	0.124	5.01E-02
Tumor necrosis factor receptor superfamily member 13B (TNFRSF13B)	0.240	0.123	5.07E-02
C-X-C motif chemokine 9 (CXCL9)	0.241	0.124	5.23E-02
Protein AMBP (AMBP)	0.245	0.126	5.29E-02
Poly [ADP-ribose] polymerase 1 (PARP-1)	0.242	0.127	5.60E-02
Interleukin-4 (IL-4)	-0.331	0.175	5.87E-02
CUB domain-containing protein 1 (CDCP1)	0.228	0.121	5.98E-02
Fibroblast growth factor 21 (FGF-21)	0.225	0.121	6.30E-02
C-X-C motif chemokine 11 (CXCL11)	0.228	0.125	6.87E-02
ADM (ADM)	0.222	0.123	7.12E-02
Natriuretic peptides B (BNP)	-0.345	0.194	7.58E-02
Heat shock 27 kDa protein (HSP 27)	0.223	0.126	7.79E-02
Galectin-4 (Gal-4)	0.216	0.123	7.86E-02
Alpha-L-iduronidase (IDUA)	0.215	0.122	7.93E-02
Interleukin-1 receptor antagonist protein (IL-1ra)	0.208	0.121	8.68E-02
Spondin-2 (SPON2)	0.202	0.122	9.82E-02

Name	Beta	Se	P
TNF-related activation-induced cytokine (TRANCE)	-0.204	0.124	9.92E-02
Interleukin-20 (IL-20)	0.339	0.210	1.07E-01
Perlecan (PLC)	0.199	0.124	1.11E-01
Tumor necrosis factor receptor superfamily member 10C (TNFRSF10C)	-0.194	0.122	1.12E-01
C-C motif chemokine 16 (CCL16)	0.198	0.124	1.12E-01
Myeloperoxidase (MPO)	0.194	0.123	1.14E-01
Growth/differentiation factor 2 (GDF-2)	-0.194	0.123	1.15E-01
Hydroxyacid oxidase 1 (HAOX1)	0.194	0.123	1.15E-01
Matrix metalloproteinase-12 (MMP-12)	0.189	0.121	1.18E-01
Interleukin-18-binding protein (IL-18BP)	0.193	0.125	1.25E-01
Tumor necrosis factor receptor superfamily member 11A (TNFRSF11A)	0.189	0.123	1.26E-01
Fibroblast growth factor 5 (FGF-5)	0.209	0.138	1.30E-01
Carbonic anhydrase 5A, mitochondrial (CA5A)	0.194	0.129	1.32E-01
Fatty acid-binding protein, adipocyte (FABP4)	0.181	0.121	1.36E-01
Osteoprotegerin (OPG)	0.186	0.125	1.37E-01
Trefoil factor 3 (TFF3)	0.180	0.122	1.41E-01
Tumor necrosis factor receptor 2 (TNF-R2)	0.181	0.126	1.51E-01
Cystatin D (CST5)	0.178	0.124	1.53E-01
C-C motif chemokine 3 (CCL3)	0.178	0.125	1.55E-01
Tissue factor pathway inhibitor (TFPI)	0.180	0.126	1.55E-01
Macrophage receptor MARCO (MARCO)	0.175	0.124	1.57E-01
Interleukin-27 (IL-27)	0.170	0.122	1.66E-01
Interleukin-1 receptor-like 2 (IL1RL2)	0.169	0.122	1.66E-01
Cathepsin L1 (CTSL1)	0.169	0.123	1.68E-01
Myeloblastin (PRTN3)	0.168	0.122	1.69E-01
Monocyte chemotactic protein 3 (MCP-3)	0.206	0.152	1.76E-01
Tissue factor (TF)	0.162	0.123	1.87E-01
Granulins (GRN)	0.158	0.122	1.96E-01
Matrix metalloproteinase-10 (MMP-10)	0.161	0.125	1.99E-01
Interleukin-8 (IL-8)	0.157	0.123	2.03E-01

Name	Beta	Se	P
Tyrosine-protein phosphatase non-receptor type substrate 1 (SHPS-1)	0.158	0.124	2.03E-01
Gastric intrinsic factor (GIF)	0.156	0.123	2.05E-01
Kallikrein-6 (KLK6)	0.150	0.123	2.26E-01
Pro-interleukin-16 (IL16)	0.150	0.125	2.29E-01
Interleukin-5 (IL-5)	-0.204	0.171	2.34E-01
Delta and Notch-like epidermal growth factor-related receptor (DNER)	-0.147	0.124	2.36E-01
Serine/threonine-protein kinase 4 (STK4)	-0.143	0.121	2.37E-01
Retinoic acid receptor responder protein 2 (RARRES2)	0.146	0.123	2.38E-01
Scavenger receptor cysteine-rich type 1 protein M130 (CD163)	0.145	0.124	2.43E-01
Tyrosine-protein kinase receptor UFO (AXL)	-0.147	0.126	2.44E-01
Plasminogen activator inhibitor 1 (PAI)	0.141	0.121	2.44E-01
Cathepsin Z (CTSZ)	0.143	0.123	2.45E-01
Neurotrophin-3 (NT-3)	-0.143	0.124	2.50E-01
Eotaxin-1 (CCL11)	0.141	0.123	2.50E-01
Lymphotactin (XCL1)	0.141	0.124	2.54E-01
C-C motif chemokine 28 (CCL28)	0.144	0.126	2.54E-01
Serpin A12 (SERPINA12)	-0.141	0.125	2.59E-01
C-C motif chemokine 24 (CCL24)	0.137	0.123	2.64E-01
Interleukin-6 receptor subunit alpha (IL-6RA)	-0.138	0.124	2.64E-01
Receptor for advanced glycosylation end products (RAGE)	0.137	0.123	2.66E-01
Interleukin-17 receptor A (IL-17RA)	0.136	0.123	2.71E-01
Melusin (ITGB1BP2)	-0.133	0.123	2.78E-01
Interleukin-17D (IL-17D)	0.132	0.122	2.81E-01
2,4-dienoyl-CoA reductase, mitochondrial (DECR1)	-0.130	0.122	2.86E-01
Azurocidin (AZU1	0.131	0.123	2.89E-01
Urokinase plasminogen activator surface receptor (U-PAR)	0.131	0.124	2.93E-01
Interleukin-17C (IL-17C)	-0.160	0.154	2.99E-01
Collagen alpha-1(I) chain (COL1A1)	-0.127	0.123	3.02E-01
Tumor necrosis factor receptor superfamily member 6 (FAS)	0.126	0.124	3.07E-01
Chitotriosidase-1 (CHIT1)	0.128	0.126	3.09E-01

Name	Beta	Se	P
Sulfotransferase 1A1 (ST1A1)	-0.123	0.123	3.18E-01
Growth hormone (GH)	-0.121	0.121	3.20E-01
Elafin (PI3)	0.123	0.124	3.23E-01
Protein S100-A12 (EN-RAGE)	-0.123	0.125	3.25E-01
Spondin-1 (SPON1)	0.122	0.125	3.30E-01
Tissue-type plasminogen activator (t-PA)	0.122	0.126	3.34E-01
Axin-1 (AXIN1)	-0.119	0.123	3.38E-01
C-C motif chemokine 15 (CCL15)	0.118	0.124	3.40E-01
Urokinase-type plasminogen activator (uPA)	0.115	0.122	3.45E-01
TNF-related apoptosis-inducing ligand receptor 2 (TRAIL-R2)	0.115	0.123	3.47E-01
Ephrin type-B receptor 4 (EPHB4)	0.113	0.124	3.63E-01
Interleukin-2 receptor subunit alpha (IL2-RA)	0.115	0.127	3.66E-01
Prolargin (PRELP)	0.109	0.121	3.69E-01
Chitinase-3-like protein 1 (CHI3L1)	0.109	0.123	3.73E-01
Leukemia inhibitory factor (LIF)	-0.196	0.229	3.91E-01
Leptin (LEP)	0.105	0.123	3.96E-01
Carboxypeptidase B (CPB1)	0.103	0.122	3.99E-01
Tartrate-resistant acid phosphatase type 5 (TR-AP)	0.103	0.124	4.08E-01
Hepatocyte growth factor (HGF)	0.106	0.128	4.10E-01
Decorin (DCN)	0.099	0.122	4.15E-01
Lymphotoxin-beta receptor (LTBR)	0.103	0.127	4.19E-01
Tumor necrosis factor receptor superfamily member 9 (TNFRSF9)	0.103	0.127	4.19E-01
Intercellular adhesion molecule 2 (ICAM-2)	-0.099	0.123	4.21E-01
A disintegrin and metalloproteinase with thrombospondin motifs 13 (ADAM-TS13)	0.097	0.121	4.23E-01
Monocyte chemotactic protein 4 (MCP-4)	0.099	0.124	4.26E-01
Caspase-8 (CASP-8)	-0.099	0.125	4.30E-01
CD40 ligand (CD40-L)	-0.096	0.122	4.30E-01
E-selectin (SELE)	0.097	0.123	4.30E-01
Interleukin-1 receptor type 1 (IL-1RT1)	-0.096	0.123	4.36E-01
Interleukin-1 receptor type 2 (IL-1RT2)	0.096	0.124	4.37E-01

Name	Beta	Se	P
Caspase-3 (CASP-3)	-0.094	0.123	4.43E-01
C-C motif chemokine 19 (CCL19)	-0.092	0.122	4.50E-01
Kidney injury molecule 1 (KIM-1)	0.093	0.123	4.51E-01
Heme oxygenase 1 (HO-1)	0.091	0.122	4.57E-01
Matrix metalloproteinase-1 (MMP-1)	0.092	0.125	4.63E-01
Tumor necrosis factor receptor superfamily member 14 (TNFRSF14)	0.090	0.124	4.68E-01
Epithelial cell adhesion molecule (Ep-CAM)	0.089	0.124	4.72E-01
Growth/differentiation factor 15 (GDF-15)	0.085	0.122	4.84E-01
T-cell surface glycoprotein CD5 (CD5)	-0.088	0.126	4.86E-01
Osteoclast-associated immunoglobulin-like receptor (hOSCAR)	0.085	0.123	4.88E-01
Superoxide dismutase [Mn], mitochondrial (SOD2)	0.085	0.123	4.89E-01
Interleukin-17A (IL-17A)	0.110	0.159	4.91E-01
Tumor necrosis factor receptor 1 (TNF-R1)	0.086	0.124	4.92E-01
Thrombospondin-2 (THBS2)	0.085	0.124	4.93E-01
TNF-beta (TNFB)	-0.084	0.123	4.94E-01
SIR2-like protein 2 (SIRT2)	-0.084	0.124	4.98E-01
Tyrosine-protein kinase Mer (MERTK)	0.085	0.125	4.99E-01
Artemin (ARTN)	-0.129	0.191	4.99E-01
Lectin-like oxidized LDL receptor 1 (LOX-1)	0.083	0.123	5.00E-01
SLAM family member 7 (SLAMF7)	0.101	0.151	5.04E-01
Interleukin-15 receptor subunit alpha (IL-15RA)	0.083	0.125	5.10E-01
Junctional adhesion molecule A (JAM-A)	-0.081	0.124	5.11E-01
Transforming growth factor alpha (TGF-alpha)	0.082	0.126	5.14E-01
Cystatin-B (CSTB)	0.078	0.121	5.20E-01
Thymic stromal lymphopoietin (TSLP)	0.417	0.668	5.33E-01
Angiopoietin-1 (ANG-1)	-0.075	0.121	5.37E-01
Proprotein convertase subtilisin/kexin type 9 (PCSK9)	-0.074	0.121	5.43E-01
Dickkopf-related protein 1 (Dkk-1)	0.075	0.123	5.45E-01
Contactin-1 (CNTN1)	-0.074	0.122	5.45E-01
Agouti-related protein (AGRP)	0.074	0.123	5.45E-01

Name	Beta	Se	P
C-X-C motif chemokine 5 (CXCL5)	-0.075	0.125	5.48E-01
Interleukin-13 (IL-13)	-0.126	0.210	5.48E-01
T-cell surface glycoprotein CD4 (CD4)	0.072	0.121	5.52E-01
Interleukin-6 (IL-6)	0.071	0.121	5.58E-01
Platelet-derived growth factor subunit A (PDGF subunit A)	-0.068	0.121	5.74E-01
Oncostatin-M (OSM)	-0.070	0.125	5.76E-01
von Willebrand factor (vWF)	-0.069	0.123	5.77E-01
C-C motif chemokine 17 (CCL17)	0.070	0.126	5.79E-01
Interleukin-18 receptor 1 (IL-18R1)	-0.068	0.123	5.80E-01
Gastrotropin (GT)	-0.071	0.129	5.82E-01
Epidermal growth factor receptor (EGFR)	-0.069	0.125	5.83E-01
Tumor necrosis factor (Ligand) superfamily, member 12 (TWEAK)	-0.067	0.123	5.87E-01
Platelet endothelial cell adhesion molecule (PECAM-1)	-0.067	0.123	5.88E-01
Interleukin-33 (IL-33)	-0.171	0.317	5.90E-01
Follistatin (FS)	0.065	0.122	5.95E-01
Tumor necrosis factor receptor superfamily member 10A (TNFRSF10A)	0.065	0.123	5.98E-01
Tumor necrosis factor ligand superfamily member 14 (TNFSF14)	-0.064	0.123	6.02E-01
Cadherin-5 (CDH5)	-0.059	0.123	6.28E-01
ST2 protein (ST2)	0.058	0.120	6.32E-01
T cell surface glycoprotein CD6 isoform (CD6)	-0.059	0.124	6.33E-01
P-selectin glycoprotein ligand 1 (PSGL-1)	0.057	0.122	6.41E-01
Polymeric immunoglobulin receptor (PIgR)	0.057	0.125	6.47E-01
P-selectin (SELP)	-0.056	0.123	6.51E-01
Pulmonary surfactant-associated protein D (PSP-D)	-0.059	0.132	6.53E-01
Lipoprotein lipase (LPL)	0.056	0.124	6.54E-01
Interleukin-20 receptor subunit alpha (IL-20RA)	-0.093	0.211	6.59E-01
C-X-C motif chemokine 16 (CXCL16)	0.055	0.125	6.60E-01
Myoglobin (MB)	0.054	0.123	6.62E-01
Integrin beta-2 (ITGB2)	-0.054	0.123	6.63E-01
C-C motif chemokine 25 (CCL25)	0.052	0.123	6.74E-01

Name	Beta	Se	P
C-C motif chemokine 23 (CCL23)	0.050	0.125	6.90E-01
Trem-like transcript 2 protein (TLT-2)	0.048	0.125	7.00E-01
Sortilin (SORT1)	0.048	0.124	7.01E-01
Low affinity immunoglobulin gamma Fc region receptor II-b (IgG Fc receptor II-b)	0.048	0.125	7.01E-01
C-C motif chemokine 22 (CCL22)	0.048	0.124	7.01E-01
Monocyte chemotactic protein 2 (MCP-2)	0.048	0.126	7.03E-01
Leukemia inhibitory factor receptor (LIF-R)	0.046	0.126	7.14E-01
C-C motif chemokine 20 (CCL20)	-0.045	0.124	7.17E-01
Aminopeptidase N (AP-N)	-0.044	0.123	7.22E-01
Insulin-like growth factor-binding protein 7 (IGFBP-7)	0.043	0.121	7.23E-01
Programmed cell death 1 ligand 1 (PD-L1)	0.044	0.124	7.24E-01
Brother of CDO (Protein BOC)	-0.043	0.121	7.25E-01
Thrombomodulin TM	0.043	0.124	7.27E-01
Proheparin-binding EGF-like growth factor (HB-EGF)	0.042	0.121	7.28E-01
C-C motif chemokine 4 (CCL4)	-0.043	0.125	7.29E-01
Insulin-like growth factor-binding protein 1 (IGFBP-1)	0.043	0.125	7.31E-01
Galectin-3 (Gal-3)	-0.042	0.124	7.32E-01
Programmed cell death 1 ligand 2 (PD-L2)	0.042	0.123	7.35E-01
Matrix metalloproteinase-2 (MMP-2)	-0.041	0.122	7.36E-01
TNF-related apoptosis-inducing ligand (TRAIL)	0.041	0.122	7.37E-01
Fractalkine (CX3CL1)	-0.042	0.124	7.38E-01
Protein-glutamine gamma-glutamyltransferase 2 (TGM2)	-0.041	0.125	7.42E-01
Latency-associated peptide transforming growth factor beta-1 (LAP TGF-beta-1)	0.041	0.124	7.42E-01
C-X-C motif chemokine 10 (CXCL10)	0.040	0.122	7.45E-01
Pappalysin-1 (PAPPA)	-0.040	0.124	7.45E-01
V-set and immunoglobulin domain-containing protein 2 (VSIG2)	-0.041	0.125	7.46E-01
Lactoylglutathione lyase (GLO1)	0.039	0.121	7.47E-01
NF-kappa-B essential modulator (NEMO)	-0.040	0.123	7.47E-01
Interleukin-18 (IL-18)	-0.040	0.123	7.47E-01
SLAM family member 5 (CD84)	0.038	0.123	7.58E-01

Name	Beta	Se	P
Osteopontin (OPN)	-0.038	0.123	7.60E-01
Transferrin receptor protein 1 (TR)	-0.036	0.120	7.65E-01
Complement component C1q receptor (CD93)	-0.036	0.123	7.69E-01
Brain-derived neurotrophic factor (BDNF)	0.039	0.133	7.71E-01
Angiopoietin-1 receptor (TIE2)	0.036	0.123	7.73E-01
Angiotensin-converting enzyme 2 (ACE2)	0.035	0.122	7.75E-01
Platelet-derived growth factor subunit B (PDGF subunit B)	-0.034	0.121	7.77E-01
Interleukin-10 (IL-10)	-0.034	0.125	7.86E-01
CD40L receptor (CD40)	-0.033	0.124	7.89E-01
Interleukin-4 receptor subunit alpha (IL-4RA)	-0.031	0.124	8.04E-01
Placenta growth factor (PlGF)	0.030	0.125	8.09E-01
Macrophage colony-stimulating factor 1 (CSF-1)	-0.030	0.129	8.19E-01
Pentraxin-related protein PTX3 (PTX3)	0.028	0.124	8.20E-01
Thrombopoietin (THPO)	0.026	0.124	8.35E-01
Proteinase-activated receptor 1 (PAR-1)	-0.025	0.124	8.41E-01
C-X-C motif chemokine 1 (CXCL1)	-0.024	0.123	8.49E-01
Vascular endothelial growth factor D (VEGF-D)	0.022	0.123	8.59E-01
Matrix metalloproteinase-3 (MMP-3)	-0.021	0.122	8.61E-01
Tumor necrosis factor ligand superfamily member 13B (TNFSF13B)	0.021	0.124	8.63E-01
Fibroblast growth factor 19 (FGF-19)	-0.021	0.123	8.67E-01
Prostasin (PRSS8)	0.020	0.122	8.73E-01
Interleukin-1 alpha (IL-1 alpha)	0.099	0.635	8.77E-01
STAM-binding protein (STAMPB)	-0.019	0.124	8.82E-01
Interleukin-12 subunit beta (IL-12B)	-0.018	0.125	8.83E-01
Interleukin-7 (IL-7)	-0.018	0.122	8.85E-01
Interleukin-10 receptor subunit beta (IL-10RB)	0.018	0.127	8.85E-01
Bleomycin hydrolase (BLM hydrolase)	-0.018	0.125	8.87E-01
Neurogenic locus notch homolog protein 3 (Notch 3)	-0.016	0.122	8.94E-01
Matrix extracellular phosphoglycoprotein (MEPE)	0.016	0.124	8.95E-01
Interleukin-2 receptor subunit beta (IL-2RB)	-0.034	0.264	8.98E-01

Name	Beta	Se	P
Fibroblast growth factor 23 (FGF-23)	-0.014	0.123	9.10E-01
Matrix metalloproteinase-9 (MMP-9)	-0.013	0.123	9.13E-01
CD166 antigen (ALCAM)	0.014	0.125	9.14E-01
C-X-C motif chemokine 6 (CXCL6)	0.012	0.127	9.22E-01
Tumor necrosis factor (TNF)	0.019	0.205	9.27E-01
Serine protease 27 (PRSS27)	-0.010	0.124	9.33E-01
Resistin (RETN)	0.009	0.122	9.39E-01
Protein delta homolog 1 (DLK-1)	-0.009	0.122	9.44E-01
Monocyte chemotactic protein 1 (MCP-1)	-0.008	0.122	9.45E-01
Beta-nerve growth factor (Beta-NGF)	-0.008	0.124	9.46E-01
Stem cell factor (SCF)	0.008	0.123	9.50E-01
Secretoglobin family 3A member 2 (SCGB3A2)	-0.007	0.123	9.58E-01
Chymotrypsin C (CTRC)	0.006	0.126	9.61E-01
Carboxypeptidase A1 (CPA1)	-0.006	0.121	9.63E-01
Peptidoglycan recognition protein 1 (PGLYRP1)	0.005	0.123	9.65E-01
Natural killer cell receptor 2B4 (CD244)	0.005	0.124	9.66E-01
Vascular endothelial growth factor A (VEGF-A)	0.005	0.123	9.69E-01
Glial cell line-derived neurotrophic factor (GDNF)	-0.004	0.126	9.72E-01
Carcinoembryonic antigenrelated cell adhesion molecule 8 (CEACAM8)	-0.004	0.122	9.74E-01
Paraoxonase (PON3)	0.004	0.122	9.75E-01
Fms-related tyrosine kinase 3 ligand (Flt3L)	0.003	0.121	9.77E-01
Bone morphogenetic protein 6 (BMP-6)	-0.002	0.121	9.86E-01
Signaling lymphocytic activation molecule (SLAMF1)	0.002	0.123	9.86E-01
Eukaryotic translation initiation factor 4E-binding protein 1 (4E-BP1)	-0.002	0.124	9.87E-01
Interleukin-10 receptor subunit alpha (IL-10RA)	-0.002	0.166	9.91E-01
